# Elimination of Senescent Macrophages by Purinergic Receptor P2RX7 Antagonist to Safeguard Against Kidney Injury and Aging

**DOI:** 10.7150/ijbs.138299

**Published:** 2026-07-30

**Authors:** Lei Tang, Letian Yang, Ruijia Zhang, Jian Li, Fan Guo, Haoyu Ye, Ping Zhou, Fei Liu, Liang Ma, Ping Fu

**Affiliations:** 1 Department of Nephrology, Institute of Kidney Diseases, West China Hospital of Sichuan University, Chengdu 610041, China.; 2 Department of Biotherapy, Cancer Center and State Key Laboratory of Biotherapy, West China Hospital of Sichuan University, Chengdu 610041, China.; 3 Department of Pediatric Nephrology and Rheumatology, Sichuan Provincial Women's and Children's Hospital / The Affiliated Women's and Children's Hospital of Chengdu Medical College. Sichuan Clinical Research Center for pediatric nephrology, Chengdu, Sichuan, 610045, China.; 4 State Key Laboratory of Quality Research in Chinese Medicines, Macau University of Science and Technology, Macau, China.

**Keywords:** kidney injury, kidney aging, macrophage senescence, P2RX7, antagonist

## Abstract

Macrophage senescence is a pathological feature in aging or diseased kidneys. However, the role of senescent macrophages in kidney injury and aging has not been fully elucidated yet. We integrated the analysis of single-cell RNA sequencing datasets and the adoptively transfusion of pretreated bone marrow-derived macrophages to investigate the role of renal macrophage senescence in kidney injury. Here, we portrayed the senescence trajectory along multiple time points in infiltrating macrophages, and observed the persistent increase of macrophage-expressed purinergic receptor P2RX7 along the senescence trajectory in injured kidneys of septic mice. Importantly, our discovered small-molecule P2RX7 antagonist strikingly improved kidney function and pathological damage, as well as mitigated macrophage senescence in septic and aging mice. Mechanistically, P2RX7 antagonist could promote the wound healing, migration and proliferation capacity of senescent reparative macrophages, thus exerting anti-inflammatory effects and repairing kidney tissues. Together, our findings illustrate the crucial participation of senescent macrophages in septic kidney injury, and offer novel therapeutic strategy via intervening P2RX7 against immunosenescence-associated kidney injury and aging.

## Introduction

Senescence is a permanent state of failing to re-enter the cell cycle in proliferating cells. It could be categorized into 'replicative senescence' and 'premature senescence'. In the field of nephrology, for instance, apart from the aging process, the senescent characteristics of parenchymal cells have also been figured out in acute kidney injury (AKI)^1^,^2^ and chronic kidney disease (CKD)^3^-^6^. Despite parenchymal cells, when involving immune cells, it is termed immunosenescence, describing a kind of innate or adaptive immune system dysfunction disturbing tissue homeostasis along life course^7^,^8^. The role of immunosenescence in different immune cells has been partly uncovered in some scenarios such as tumor microenvironment^9^,^10^, neurodegenerative^11^ and cardiometabolic diseases^12^. Across all types of immune cells, macrophages have received considerable scrutiny due to their mighty immunomodulating function during diseases and aging^13^. Regarded as an acute inflammatory disease, septic AKI is majorly recognized as a hyperactive innate immune response and subsequent 'cytokine storm' after pathogen infection^14^*.* The premature macrophage senescence is also involved in septic AKI and its role is worth further exploration.

The function of macrophages is a critical determinant on organ pathology and regeneration, shaped by a dynamic balance between their different phenotypes driven by opposing cytokine signals, among which the type 2 cytokines such as IL4 or IL13 could induce the differentiation of an alternatively activated macrophage (AAM, also known as M2a) phenotype displaying characteristics of highly expressed CD206, Arg1 and Chil3^15^. Studies have shown that such type 2 cytokine-stimulated macrophages could adopt a pro-resolving phenotype that promoted tubule repair in the context of ischemia/reperfusion (I/R)-associated AKI^16^. Previous studies have pinpointed the decline in type 2 cytokine signaling pathway, as well as the relative cell number of CD206^pos^ macrophage population as crucial triggers of physical dysfunction during aging^17^,^18^. Therefore, we hypothesize that cellular senescence may compromise the function of these reparative macrophages during kidney injury, eventually contributing to the tissue impairment and aging.

The purinergic receptor P2RX7 is a classic pro-inflammatory drug target highly expressed on immune cells. In kidney, inhibiting tubular epithelial cell- or T cell-expressed P2RX7 could rescue the inflammatory response and renal dysfunction in I/R AKI^19^,^20^. In our previous studies, we have discovered novel and potent small-molecule P2RX7 antagonists against AKI and CKD, respectively^20^,^21^. However, the roles of targeting P2RX7 to protect macrophage senescence in septic AKI and aging is unclear.

Herein, we first integrated the analysis of single-cell RNA sequencing (scRNA-seq) datasets and the adoptively transfusion of pretreated bone marrow-derived macrophages (BMDMs) to confirm the role of renal macrophages senescence in septic AKI. As a potential therapeutic strategy, our small-molecule P2RX7 antagonist could safeguard against septic AKI and aging, probably through mitigating the underlying immunosenescence of macrophages exhibiting **reparative phenotypes**.

## Materials & Methods

### Animals

The protocol of the animal research was reviewed and authorized through the Experimental Animal Ethics Committee of West China Hospital, Sichuan University (20230709001). We used the ARRIVE checklist when writing our report^23^. Six- to- eight-week-old male C57/BL6J mice were randomly grouped (n = 6). The septic AKI group was provided with an intraperitoneal injection of 10mg/kg lipopolysaccharide (LPS, L8880, Solarbio) or underwent cecal ligation and perforation (CLP). Methods of establishing CLP was previously described^24^. In the meantime, the control group was provided with an equal volume of 0.9% saline or had sham surgery respectively. P2RX7 antagonist 14A (P2RX7A) produced in house was administered intraperitoneally at a dose of 20mg/kg was given 30min before as well as 8hrs after LPS injection or CLP^21^. 16hrs after injecting LPS or undergoing CLP, the mice were sacrificed.

Besides, we used 13A, another type of P2RX7A whose efficacy has been confirmed in mice with chronic kidney disease to treat aged mice^22^. 18-month-old male C57/BL6J mice were randomly grouped (n = 6). P2RX7A produced in house was administered at a dose of 1mg/kg twice a week by gavage for three months. At the age of 21 months old, mice were sacrificed. Three-month-old mice were used as their younger counterparts.

The histological change, kidney function and the expression of senescence signatures were measured using their urine, serum and kidney tissue.

### Cell culture

Murine BMDMs were obtained from three- to- four-weeks-old male mice on C57BL/6J background. Briefly, bone marrow cells were flushed from the tibia and femurs and then cultured in complete media containing DMEM supplemented with 10% fetal bovine serum (FBS), and 1% penicillin-streptomycin-amphotericin B solution and differentiated with 20ng/ml mouse M-CSF (Z03275, GenScript) for seven days in 37°C and 5% CO_2_ incubator. Mature BMDMs were pretreated with 2.5μM P2RX7A for 1hrs, then stimulated with 100ng/ml LPS for 3hrs and 5μM adenosine triphosphate (ATP) (MB3157, Meilunbio) for 1hrs.

Human monocyte leukemia cell line THP-1 was bought from ATCC. The cells were cultured in RPMI 1640 medium supplemented with 10% FBS at 37°C in a 5% CO_2_ incubator. To transform THP-1 monocytes into adherent macrophages, 100ng/ml phorbol 12-myristate 13-acetate (P6741, Solarbio) was given for 48hrs. Likewise, THP-1 cells were pretreated with 2.5μM P2RX7A for 1hrs, then stimulated with 100ng/ml LPS for 24hrs and 5μM ATP for 1hrs.

### Adoptively transfusion of BMDMs

Murine BMDMs isolated from healthy or septic AKI mice were cultured as before. Mature BMDMs were pre-treated with 15μM Fisetin and 2.5μM P2RX7A for 24hrs. Pre-treated BMDMs were digested using trypsin and resuspended in PBS (10^6^ cells in 100μL). Before transfusion, 200μL clodronate liposomes (ClodronateLiposomes.org) or liposome-encapsulated PBS were injected into the tail vein of mice for the elimination of macrophages from myeloid origin. After 24hrs, 100μL pre-treated BMDM suspensions or PBS were injected into the tail vein of mice. After another 24hrs, mice were challenged with septic AKI models.

To make sure that the injected BMDMs could successfully function in the kidneys, we labelled them with lipophilic membrane tracers DiR (UElandy, D4006) at the concentration of 5μM. After 30 min incubation in darkness at 37°C, BMDMs were washed using PBS twice to remove free dye solutions. 24hrs after the administration of DiR-labelled BMDMs, their presence in kidneys were visualized under fluorescence imaging (Bruker Spectral Instrumental Imaging System). Excitation and emission wavelengths were set at 830 nm and 750 nm respectively.

### Flow cytometry

For the measurement of peripheral blood, red blood cell lysis buffer (BL503A, Biosharp) was used to turn mouse blood into single-cell suspensions (1:10) at RT for 5min and was neutralized using 2% FBS 1640. The cell suspension was centrifuged at 500xg in 4°C for 6min and single cells were blocked in anti-CD16/CD32 (1:100 in PBS, 553131, BD Biosciences). For cell surface staining, single cell suspensions were first incubated in Zombie Violet™ Fixable Viability Kit (423113, BioLegend) at RT for 15min, and subsequently in APC-anti-CD11b (101212, BioLegend), FITC-anti-Ly6c (128005, BioLegend) at RT for 15min. For intracellular Ki-67 staining, single cell suspensions were fixed and permeabilized using True-Nuclear™ Transcription Factor Buffer Set (424401, BioLegend) and then incubated with primary antibody anti-Ki-67 (1:500, HA721115, Huabio,) at 4°C for 1hr and PE-Cy7-conjugated secondary antibody (bs-0295G, Bioss) at RT for 45min.

For the measurement of the kidney tissue, digestion buffer (2% FBS 1640) containing 400μg/ml collagenase D (11088866001, Roche) and 10μg/ml DNaseI (11284932001, Roche) at 37°C for 30min with shaking. The digested cell suspension was filtered using 70μm strainer. Single cells were resuspended in 4ml 40% percoll (17089102, Cytiva) and further mounted over 3ml 70% percoll. Single-cell suspension was centrifuged at 900xg in RT for 20min. After careful aspiration of the supernatant, cell pellets were resuspended in 2% FBS 1640 to wash out percoll particles. The procedure of blocking, incubation of Zombie, cell surface staining of FITC-anti-CD45 (103108, BioLegend), APC-anti-CD11b and PE-anti-F4/80 (123110, BioLegend), as well as intracellular staining of p16 (ab211542, abcam), p21 (HA722065, Huabio) and Ki-67 primary antibodies and PE-Cy7-conjugated secondary antibody were the same as those of peripheral blood measurement.

For the measurement of BMDMs, after digested using trypsin, cells were blocked, incubated with Zombie, APC-Cy7-anti-CD11b (101225, BioLegend) and PE-anti-F4/80. Following fixation and permeabilization, cells were incubated with APC-anti-CD206 (E-AB-F1135E, Elabscience) and FITC-anti-BrdU (364103, BioLegend) at 4°C overnight. Then, the aforementioned intracellular staining of p21 and Ki-67 primary antibodies, as well as PE-Cy7-conjugated secondary antibody were performed.

The samples were resuspended in cell staining buffer and further acquired on CytoFLEX (BECKMAN COULTER) and analyzed with Flow Jo 10 (Treestar, Ashland, OR).

### Wound healing assay

Several scratches were made by 20µl micropipette tips in 6-well culture plate with mature BMDMs. Plates were washed with PBS to remove non-adherent and dead cells. 0, 16 and 20hrs after the scratches were made, cells along the same scratches were captured at 40× magnification under ECLIPSE Ts2R-FL research microscopy (Nikon Instruments) utilizing the NIS-Elements D 5.21.00 software.

### Transwell migration assay

Transwell inserts with pore size of 8 µm (3422, Corning) were placed in the corresponding 24-well culture plate. Lower chamber was filled with 600µl DMEM containing 20% FBS, 20ng/ml IL4 and IL13 (214-14-20UG and 210-13-10UG, PeproTech), while 5×10^4^ digested mature BMDMs were loaded on the apical side of the chamber in total volume of 200µl DMEM containing 1% FBS with or without P2RX7A. Cells were incubated for 48hrs at 37⁰C and 5%CO_2_.

Then, cells were washed with PBS, fixed for 15 min at RT with 600µl 4% paraformaldehyde (PFA) (BL539A, biosharp), and stained with 600µl 0.1% crystal violet (BL802A, biosharp) for 45 min at RT protected from light. Excess cells were removed from the apical side with a sterile swap. Migrated cells on the lower surface were captured at 100× magnification under ECLIPSE Ts2R-FL research microscopy.

### Transdermal glomerular filtration rate measurement

The transdermal GFR (tGFR) system is designed to assess kidney function by measuring the clearance rate of a fluorescent tracer agent. It consists of three key components: the tGFR sensor, the tGFR monitor, and FITC-Sinistrin (MediBeacon, Creve Coeur). The system operates by transdermally recording the fluorescence intensity of FITC-Sinistrin over time via a skin-placed sensor. The tGFR sensor captures data at a rate of 2.5 readings per second, and the tGFR monitor subsequently computes and displays the average session tGFR reading. The mice were shaved one day in advance. On the day of measurement, the tGFR sensor was placed on the mouse's skin, then FITC-Sinistrin was injected via the tail vein (7mg/100g body weight). During the measurements, the mice were allowed to move freely to ensure a natural behavioral state. 120 min later, the recording was read and analyzed using MediBeacon Studio V2 software (MediBeacon, Creve Coeur). The tGFR was calculated by analyzing the concentration time curves of FITC-Sinistrin.

### Single cell RNA-sequencing analysis

The processed data of scRNA-seq of mouse renal cells obtained at multiple time points following LPS administration was downloaded from GEO (https://www.ncbi.nlm.nih.gov/, GSE151658), including 3216 immune cells. The raw count matrix of scRNA-seq of human peripheral blood mononuclear cells (PBMCs) isolated from patients with sepsis was downloaded from Single Cell Portal (https://singlecell.broadinstitute.org/single_cell, SCP548). To save calculation time, we merely extracted the data of Bac-SEP and ICU-SEP cohort and randomly selected three healthy controls, resulting in 24583 cells included in the analysis. Raw scRNA-seq data underwent standard work flow using R package Seurat (v4.4.0), including quality control, normalization, integration, and dimension reduction. Package SingleR was used to annotate each cluster automatically. Fold changes of genes were measured using function *FindMarkers()*, then GSEA was performed according to log-fold change using package clusterProfile (v4.10.0) and fgsea (v1.28.0). Pseudotime trajectory analysis employing package monocle (v2.30.0), was conducted. Fridman, SenMayo, Reactome and CellAge were used as reference for the enrichment of senescence gene signatures.

### Statistical analysis

The results are presented as mean ± SD for at least three repetitions for cell samples and six repetitions for animal experiments. In GraphPad Prism 9.0 (GraphPad Software, San Diego, CA, USA), the data was first tested to find out whether it met the assumption of normality assessed by the Shapiro-Wilk test. Then, Mann-Whitney U test (nonparametric data) or two-tailed Student's t-test (parametric data) was utilized to analyze the differences in statistics between two groups, and one-way ANOVA with a post hoc Bonferroni test, Kruskal-Wallis multiple comparisons test was implemented to analyze differences among more than two groups. Statistical significance was recognized as p-value < 0.05.

## Results

### Mapping the monocytic origin of renal macrophage senescence in septic AKI

**Figure 1:**
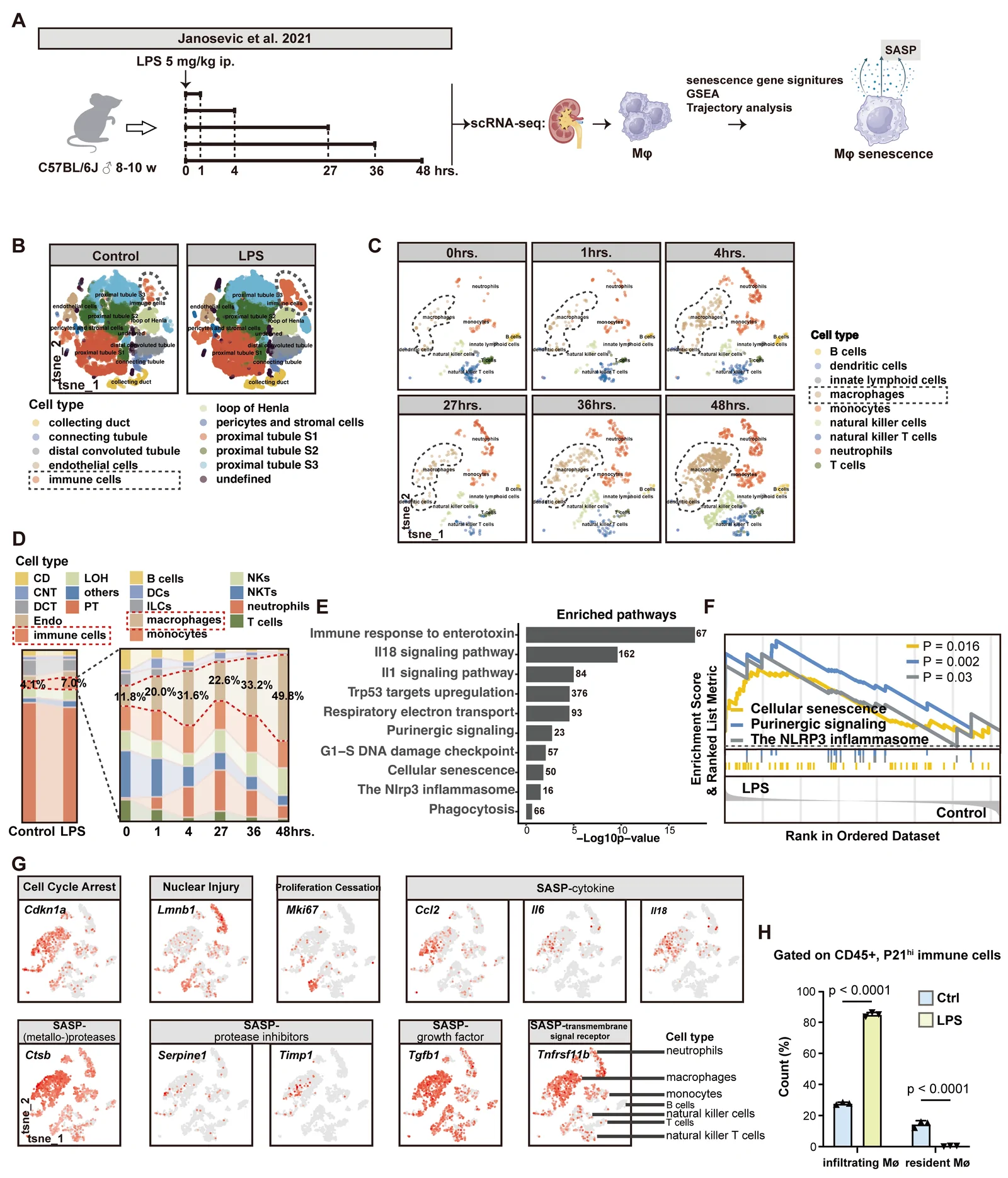
** Mapping the senescence features of renal macrophages in septic AKI.** (A) The following analysis (B-E) utilized single-cell transcriptomic data performed in murine kidneys at multiple time points after LPS administration by Janosevic *et al*. 2021 (GSE151658). (B) The tSNE embedding of single cells from murine kidneys under healthy and septic conditions, colored by cluster identity. (C) The tSNE embedding of immune single cells identified utilizing the marker *Ptprc* across multiple time points in septic AKI, colored by cluster identity. (D) The proportion of major cell types as well as immune cell subtypes changed with the development of septic AKI. (E-F) GSEA enrichment results of the differential gene expression patterns in macrophages changed in septic AKI (a combination of 1, 4, 16, 27, 36 and 48hrs) compared to healthy control (0hrs), , especially in 'cellular senescence', 'purinergic signaling' and 'NLRP3 inflammasome'. (G) The feature plot of *Cdkn1a*, *Lmnb1*, *Mki67* and genes encoding several types of senescence-associated secretory phenotypes in SenMayo (PMID: 35974106) expressed on immune cells from the injured kidney of septic mice. (H) Quantification of p21^pos^ resident macrophages (CD11b^lo^ F4/80^hi^) and p21^pos^ infiltrated macrophages (CD11b^hi^ F4/80^lo^) in the kidney of septic mice (n = 3). CD, collecting duct. CNT, connecting tubule. DCT, distal convoluted tubule. LOH, loop of Henla. DC, dentritic cell. NK, natural killer cell. ILC, innate lymphoid cell. SASP, senescence-associated secretary phenotype.

To explore the features of immune cells in septic AKI, we first reanalyzed the scRNA-seq of mouse renal cells obtained at multiple time points after LPS administration (Figures 1A-C)^25^. Septic AKI was associated with an increase in the proportion of immune cells, as well as the alteration in its composition. Macrophages increased consistently along the time and eventually comprised the largest proportion of immune cells (from 11.8% to 49.8%) (Figure 1D).

Having established a broad overview of renal immune cells in septic AKI, we further extracted macrophages and explored its features in detail. GSEA results revealed that the LPS administration led to the enrichment in immune responses, inflammation ('IL-1, IL-18 signaling pathway activation' and 'NLRP3 inflammasome') and energy metabolism ('respiratory electron transport' and 'purinergic signaling') in macrophages. Notably, LPS-stimulated macrophages also exhibited patterns consistent with 'cellular senescence', 'p53 targets upregulation' and 'G1-S DNA damage' (Figures 1E-F). A more evident senescent status of macrophages across all types of immune cells was confirmed in the injured kidneys of septic mice. Levels of *Lmnb1* and *Mki67* were comparatively lower in macrophages than other cell types in septic mice, indicating the more severe nuclear membrane instability and proliferation cessation respectively. At the same time, the expression of *Cdkn1a*, representative of cell cycle arrest, as well as genes encoding several types of senescence-associated secretary phenotypes (SASPs) was much higher in macrophages (Figure 1G).

Given that both tissue-resident and infiltrated macrophages exist in kidney tissue^26^, we further explored the origin of senescent macrophages in septic AKI using flow cytometry (Figure S1A). It is notable that p21^pos^ CD45^pos^ immune cells were largely constituted of CD11b^hi^ F4/80^lo^ monocyte-derived macrophages (~80%) rather than CD11b^lo^ F4/80^hi^ resident ones (<1%)^27^ under the septic condition, suggesting the monocytic origin of senescent renal macrophage in septic AKI (Figure 1H, Figure S1B).

Therefore, we further employed the scRNA-seq of human PBMCs isolated from patients with sepsis for confirmation (Figure S2A)^28^. Accounting for almost a half of the whole PBMCs, monocytes could be clustered into six subgroups (Figures S2B-C), among which subcluster-2 (*C1Q^hi^* monocytes) experienced the most obvious increase (from 15.9% to 25.9%, Figure S2D). The senescent state of all monocyte subclusters could be generally confirmed through their enrichment in senescence gene sets, and *C1Q^hi^* monocytes presented relatively stronger senescence characteristics (Figures S2E-H). As matter of fact, *C1Q^hi^* monocytes significantly engaged in the monocyte-to-macrophage transition^29^, probobaly accouting for the origin of senescent macrophages in the kidney tissue. In all, the scRNA-seq of PBMCs supplemented the aforementioned results that senescent macrophages in kidneys, largely generated from circulating monocytes, may play a role in septic AKI.

### Elimination of senescent macrophage alleviated kidney injury of septic mice

**Figure 2:**
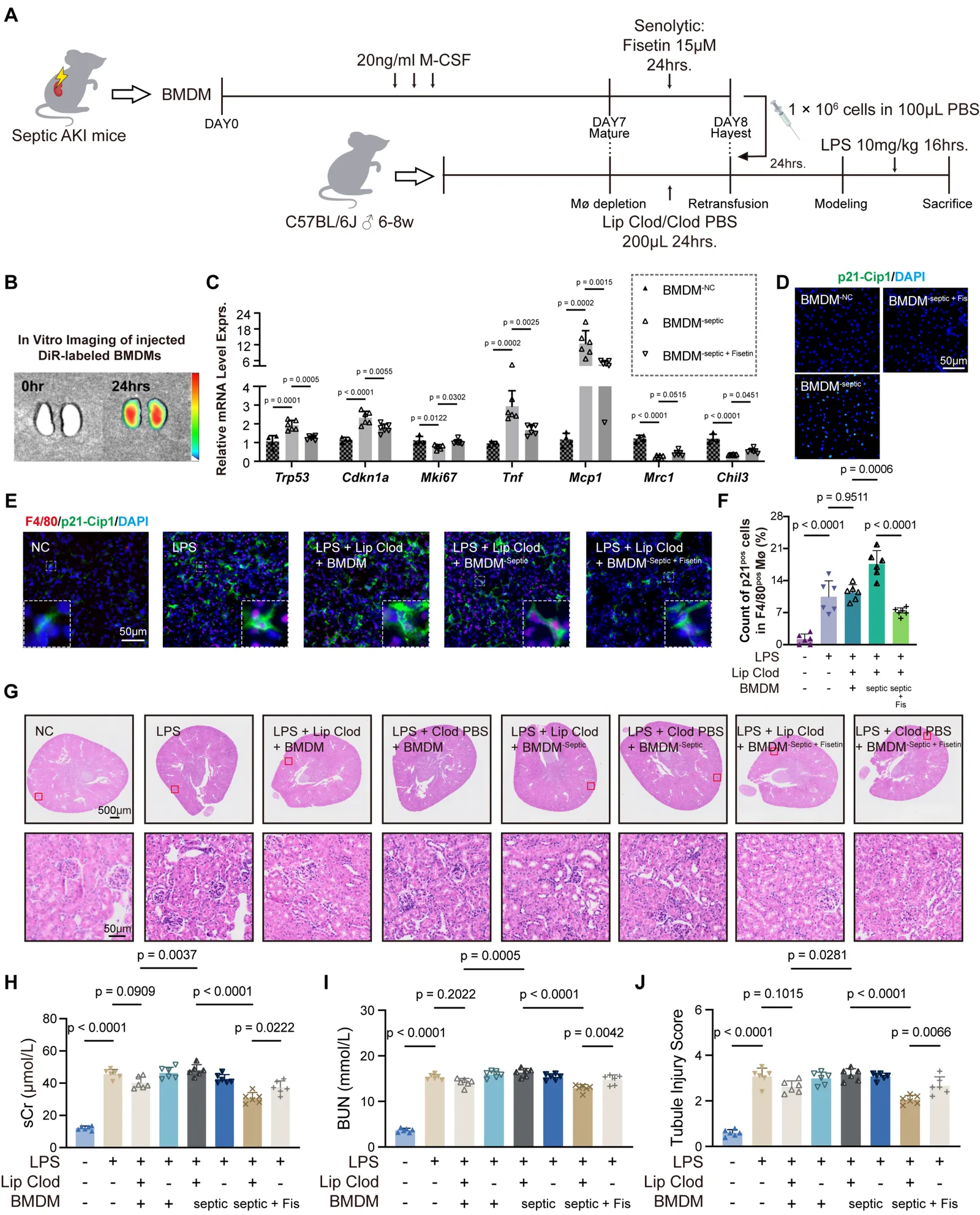
** Adoptive transfusion of BMDMs pre-treated with senolytics targeted renal macrophage senescence of monocytic origin in septic AKI.** (A) The schematic diagram of the study: BMDMs were isolated from the donor septic AKI mice and then pre-treated with senolytic (Fisetin). After the macrophage ablation, the pre-treated BMDMs were adoptively transplanted into the recipient septic AKI mice. (B) The *in vitro* fluorescence imaging of the freshly separated kidneys 24hrs after the injection of DiR-labelled BMDMs. (C-D) The alteration in immunofluorescence staining and mRNA expression levels of senescence, pro-inflammatory and pro-resolving markers in BMDMs isolated from donor septic AKI mice pre-treated with the senolytic Fisetin. (E-F) The alteration in immunofluorescence of p21 in F4/80 identified macrophages in kidney sections after the retransfusion with BMDMs isolated from donor septic AKI mice pre-treated with Fisetin (200×). (G-J) The alteration in SCr and BUN level, tubule injury score and H&E staining (1× and 200×) of recipient septic AKI mice after the retransfusion with BMDMs isolated from donor septic AKI mice pre-treated with the senolytic Fisetin (n = 6). Lipo Clod, clodronate liposomes. Lipo PBS, liposome-capsuled PBS.

To further determine the role of senescent macrophages in septic AKI, the BMDMs were isolated from the donor septic AKI mice (BMDM^-septic^) and then pre-treated with senolytic (Fisetin) to eliminate senescent BMDMs (BMDM^-septic + Fis^). After the macrophage ablation performed by clodronate liposomes, the pre-treated BMDMs were adoptively transplanted into the recipient septic AKI mice (Figure 2A). 24hrs after the injection of the DiR-labeled BMDMs, significant fluorescence was observed in the freshly separated kidneys using the *in vitro* imaging, suggesting that the transplanted BMDMs could successfully arrive and function in the kidneys (Figure 2B). The procedures of macrophage ablation and adoptive transplantation of BMDMs without pre-treatment are seemed to partially mitigate kidney injury of septic AKI mice, but the results did not reach the statistical significance (Figure S3). The BMDMs from septic AKI mice expressed the elevated levels of cell cycle arrest (*Trp53* and *Cdkn1a*) and pro-inflammatory (*Mcp1* and *Tnf*) markers, as well as the reduced levels of proliferative (*Mki67*) and pro-resolving (*Mrc1* and *Chil3*) markers. When administered with senolytic drug Fisetin, such expression pattern reversed (Figures 2C-D). As expected, transfusion of BMDM^-septic + Fis^ could decrease the expression of p21 in F4/80 marked renal macrophages of recipient septic AKI mice (Figures 2E-F). Furthermore, reno-protective effect could be observed, presented by attenuated serum creatinine (SCr) and blood urea nitrogen (BUN) levels, as well as improved renal tubular injury, including flattening of epithelial cells and expansion of lumen, compared to those with transfusion of BMDM^-septic^ which aggregated kidney impairment in septic AKI mice (Figures 2G-J). As a critical step in macrophage transplantation, the preparation of recipient mice with clodronate liposomes to ablate macrophages is necessary since the mere infusion of pre-treated BMDMs alone without albation could not achieve the same therapeutic effect (Figures 2G-J). Therefore, targeting senescent renal macrophages of monocytic origin could be a potential therapeutic strategy for septic AKI.

### Senescence caused the tissue-repaired dysfunction of renal reparative macrophages during septic AKI

**Figure 3:**
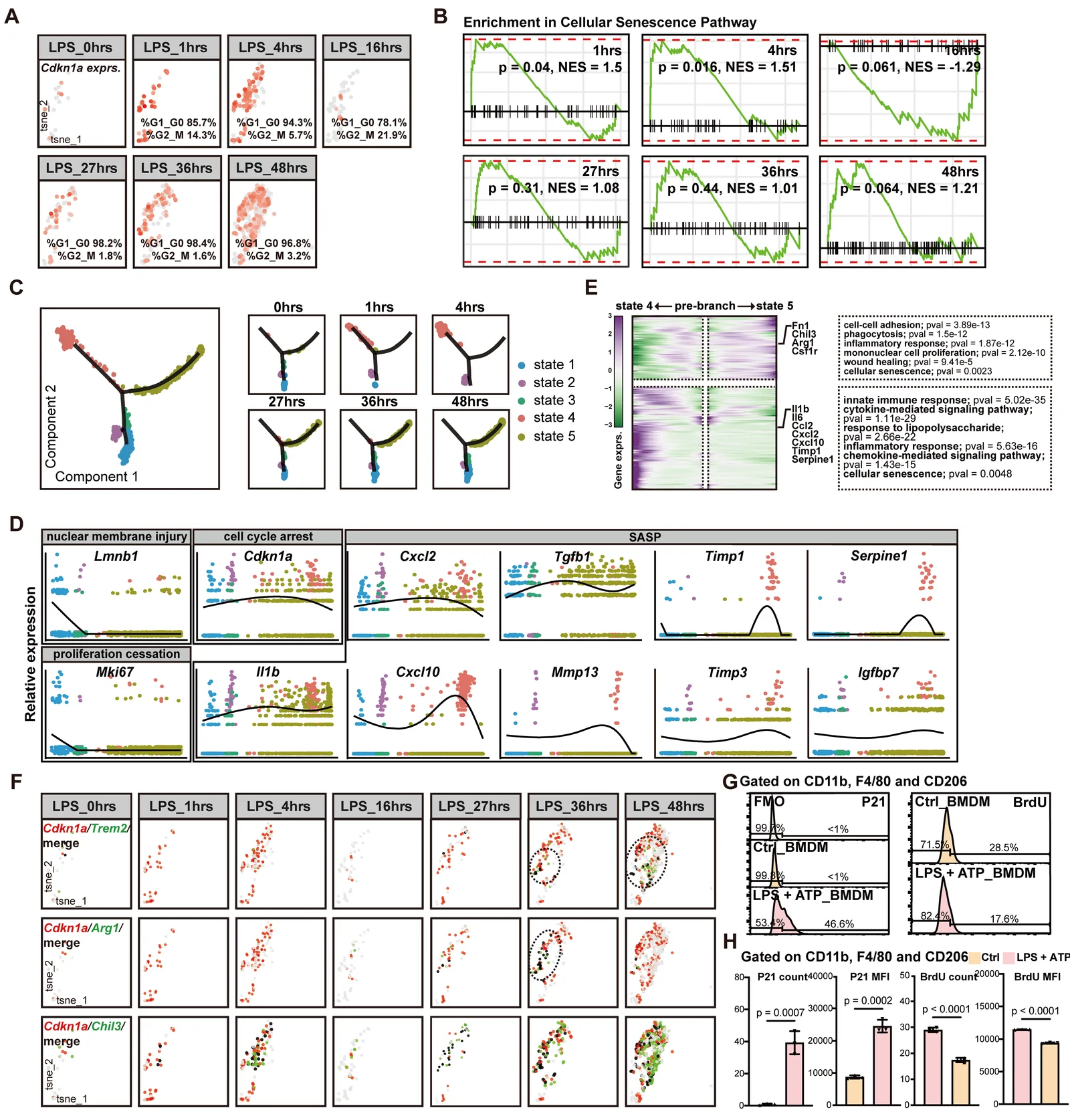
** The effect of renal macrophages senescence on its function during septic AKI.** (A-B) The feature plot of *Cdkn1a*, the results of cell cycle analysis and GSEA plot of cellular senescence pathway at multiple time points after the LPS stimulation. (C) The trajectory of renal macrophages from state 1-3 cells to state 4 or state 5 cells and the constitution of each state by different time points during septic AKI in the pseudotime analysis. (D) The feature plots of different types of senescence gene signatures in renal macrophages along the trajectory during septic AKI. (E) The heatmap of differential expressed genes and the results of GO enrichment along the trajectory during septic AKI. (F) The co-expression of *Cdkn1a* and pre-resloving macrophage markers *Trem2*, *Arg1* and *Chil3* at multiple time points after the LPS stimulation. (G-H) The quantification of p21^pos^ or BrdU^pos^ CD206^pos^ CD11b^pos^ F4/80^pos^ BMDMs under the stimulation of LPS and ATP (n = 3).

To gain insights into the detailed senescence pattern of renal macrophages in septic AKI, we measured the senescence feature of different time points after the LPS administration using scRNA-seq. The quantification of *Cdkn1a* expression, cell cycle analysis and GSEA enrichment suggested a constantly changed senescence condition, that macrophage senescence first occurred at the 1-4hrs and alleviated from 4hrs on but aggregated at 36-48hrs (Figures 3A-B). Compared with *Cdkn1a^neg^* macrophages, *Cdkn1a^pos^* senescent ones significantly enriched in the up-regulated pathways related to inflammatory response and DNA damage, and down-regulated pathways concerning phagocytosis, endocytosis, wound healing, mononuclear cell proliferation and tissue remodeling (Figure S4).

The pseudotime analysis revealed a transition of macrophages from state 1-3 to state 4 or state 5 along septic AKI development. State 4 and State 5 were mainly constituted of cells from 1-4hrs and 27-36hrs respectively (Figure 3C). Senescence gene signatures altered correspondingly in state 4 and state 5, suggesting that the transition represented two distinct senescence trajectories respectively peaking at 4hrs and 48hrs during the development of septic AKI (Figure 3D). Apart from 'cellular senescence', the trajectory towards 4hrs enriched in 'inflammatory response' and 'cytokine/chemokine signaling pathway', while the trajectory towards 48hrs enriched in 'phagocytosis', 'monocular cell proliferation' and 'wound healing' (Figure 3E). Therefore, the senescence of renal macrophages presented as the secretion of pro-inflammatory SASPs in the acute phase and dysfunction in tissue repair in the later stage of septic AKI. A group of macrophages co-expressing* Cdkn1a* and reparative markers *Trem2*, *Arg1*, *Chil3* confirmed our assumption that senescence of renal macrophages may affect its repair capability, probably causing the consistent kidney injury (Figure 3F).

Based on the results from scRNA-seq, we used flow cytometry to further verify the senescence state of pre-solving CD206^pos^ BMDMs under the stimulation of LPS and ATP. It is notable that the quantification of p21^pos^ CD206^pos^ CD11b^pos^ F4/80^pos^ BMDMs increased significantly, accompanied by the decrease in BrdU^pos^ ones in the 'LPS + ATP' group (Figures 3G-H), revealing the impaired proliferation in senescent CD206^pos^ reparative macrophages. Taken together, the above results facilitated us in getting insight into the influence of renal macrophages senescence on its function during septic AKI.

### Purinergic receptor P2RX7 was associated with renal macrophage senescence in septic AKI

**Figure 4:**
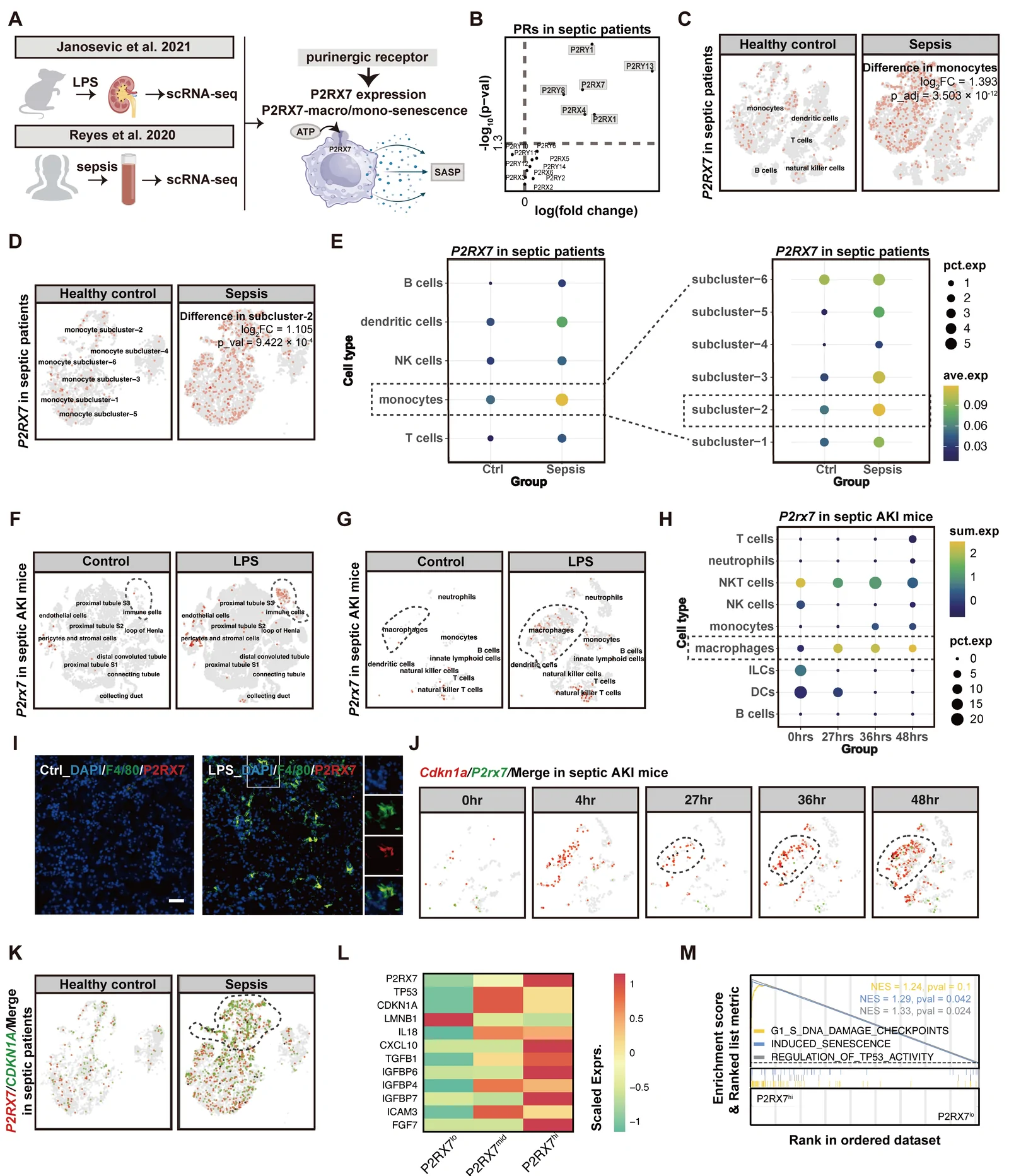
** Purinergic receptor P2RX7 was associated with renal macrophage senescence in septic AKI.** (A) The following analysis utilized single-cell transcriptomic data in murine kidneys performed at multiple time point after LPS administration by Janosevic *et al*. 2021 and in PBMCs from healthy control and sepsis patients by Reyes *et al*. 2020. (B) The relative expression of purinergic receptor family in septic patients compared to healthy control. (C-E) The feature plot and quantitation of *P2rx7* in major cell types and by immune cell types of murine kidneys under healthy and septic conditions. (F-H) The feature plot and quantitation of *P2RX7* in major cell types of human PBMCs and monocyte subclusters under healthy and septic conditions. (I) The alteration in immunofluorescence of P2RX7 (red) in F4/80 (green) identified macrophages in the kidney sections of septic AKI mice; scale bar = 50μm. (J) The co-expression pattern of *P2rx7* and *Cdkn1a* in renal macrophages from septic AKI mice at multiple time points after the LPS administration. (K) The co-expression pattern of *P2RX7* and *CDKN1A* in monocyte subclusters from human PBMCs under healthy and septic conditions. (L-M) The differential expression pattern of senescence gene signatures in *P2RX7^hi^, P2RX7^int^*, and *P2RX7^lo^* septic patients.

Having figured out the senescence feature of renal macrophages in septic AKI, we tried to explore new therapeutic strategies directed against this phenotype. Since there was a significant enrichment in 'purinergic signaling' together with 'cellular senescence' in mononuclear phagocytic cells during sepsis, we then screened the whole purinergic receptor family to find a potential target for macrophage senescence in septic AKI (Figure 4A). Of all purinergic receptors,* P2RX7* was among the top three whose expression significantly increased in monocytes of patients with sepsis (Figure 4B). Its increase was the most obvious in *C1Q^hi^* monocytes (Figures 4C-E). In the scRNA-seq of murine kidneys, LPS administration resulted in a notable increase in the expression of *P2rx7* in immune cells, among which macrophage was the only cell type that present a consistent rise in *P2rx7* along the time (Figures 4F-H). The immunofluorescence co-staining F4/80 and P2RX7 in the kidneys of the control and septic AKI mice also comfirmed the increased P2RX7 level in renal macrophages (Figure 4I).

At the same time, a co-expression pattern of *P2RX7* and *CDKN1A* could be seen both in renal macrophages and blood monocytes during sepsis, especially at the later stage of the disease course (Figures 4J-K). Furthermore, we categorized patients with sepsis according to the* P2RX7* expression in *C1Q^hi^* monocytes. As the *P2RX7* level increased in septic patients, the senescence markers changed apparently in the *P2RX7^int^* and *P2RX7^hi^* groups (Figure 4L). Compared to *P2RX7^lo^* group, *P2RX7^hi^* group expressed higher levels of senescence gene signatures. The differential expression pattern was enriched in 'G1_S DNA damage', 'TP53 activity' and 'induced senescence', indicating that P2RX7 may affect septic AKI through the senescence of mononuclear phagocytic cells (Figure 4M).

### Our discovered small-molecule P2RX7 antagonist alleviated kidney injury and eliminated renal senescentic macrophage in septic mice

**Figure 5:**
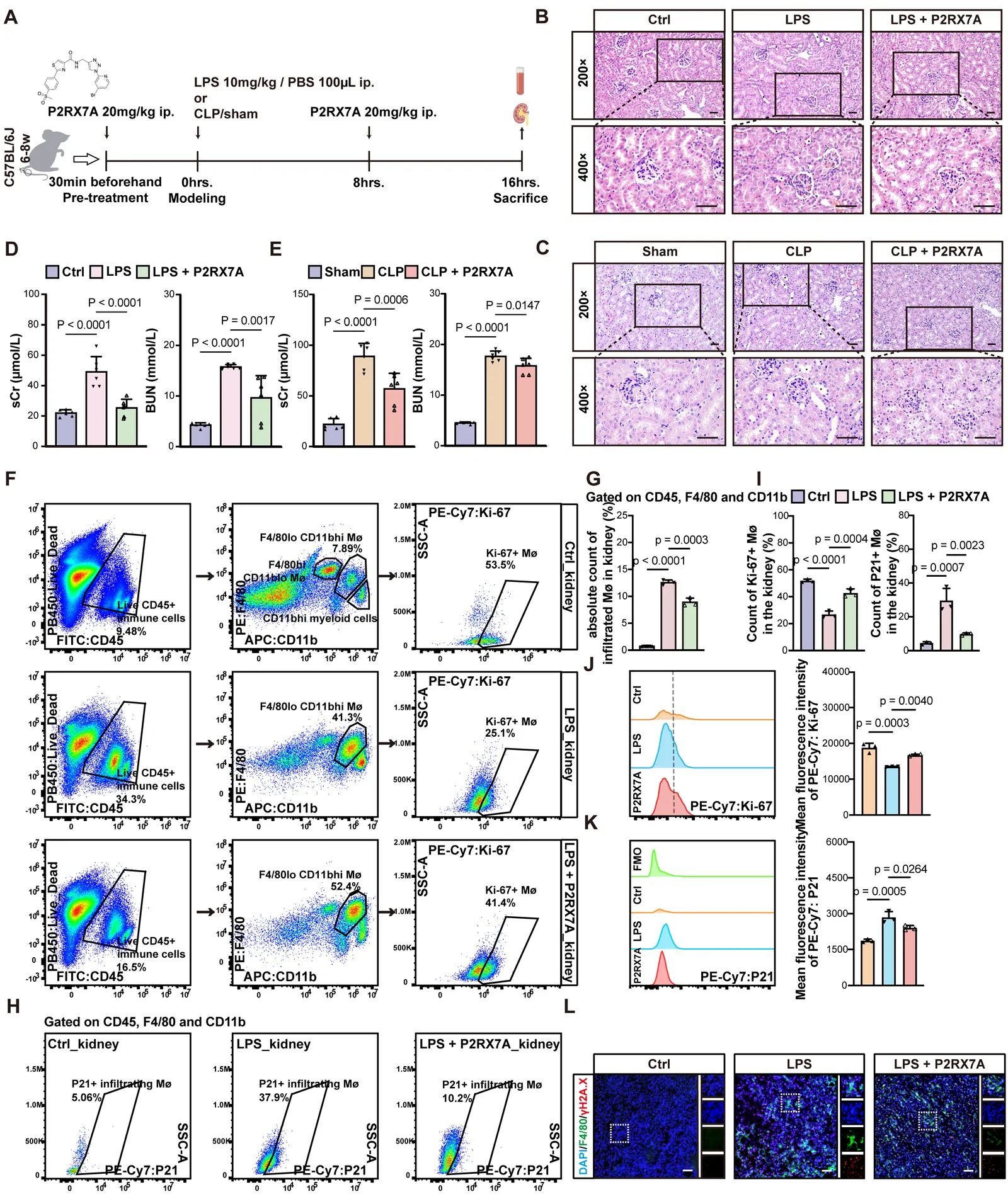
** Small-molecule P2RX7 antagonist alleviated kidney injury and renal macrophage senescence in septic mice.** (A) Schematic diagram of intervening septic AKI mice with P2RX7A. (B-E) The alteration in SCr and BUN level, H&E staining (200× and 400×) of septic AKI mice after the administration of P2RX7A (n = 6) ; scale bar = 50μm. (F, H) The gating strategy of flow cytometry measuring the number of p21^pos^ and Ki-67^pos^ CD45^pos^ CD11b^hi^ F4/80^lo^ infiltrating renal macrophages in septic AKI. (G) The alteration in the count of CD45^pos^ CD11b^hi^ F4/80^lo^ infiltrating macrophages in the kidneys of septic AKI mice after the administration of P2RX7A (n = 3). (I-K) The alteration in the quantification of p21^pos^ and Ki-67^pos^ CD45^pos^ CD11b^hi^ F4/80^lo^ infiltrating macrophages in the kidneys of septic AKI mice after the administration of P2RX7A (n = 3). (L) The alteration in immunofluorescence of γH2A.X (red) in F4/80 (green) identified macrophages in the kidney sections of septic AKI mice after the administration of P2RX7A; scale bar = 50μm.

Our discovered small-molecule P2RX7 antagonist 14A (P2RX7A)^21^ was used to investigate the role of antagonizing P2RX7 in the renal macrophage senescence of septic AKI (Figure 5A). There was no death in two classic models of septic AKI. P2RX7A significantly attenuated the histological damage of injured kidney, and improved kidney function (Figures 5B-E, Figure S5A). The results of flow cytometry revealed a decrease in the infiltration of the whole CD45^pos^ immune cells, and the absolute number of CD45^pos^ CD11b^hi^ F4/80^lo^ monocyte-derived macrophages almost by a half after the administration of P2RX7A in the kidneys of septic AKI mice (Figures 5F-G). More importantly, the quantification of p21^pos^ CD45^pos^ CD11b^hi^ F4/80^lo^ infiltrating renal macrophages was reduced, while those whose markers expressed as Ki-67^pos^ CD45^pos^ CD11b^hi^ F4/80^lo^ was restored (Figures 5H-K). Immunofluorescence co-staining F4/80 and γH2A.X also illustrated a decreased level cellular senescence in the renal macrophages of septic AKI mice using P2RX7A as the treatment (Figure 5L). At the same time, P2RX7A significantly lowered the number of CD11b^pos^ Ly6C^pos^ monocytes in the peripheral blood of septic AKI mice. The quantification of non-senescent Ki-67^pos^ CD11b^pos^ Ly6C^pos^ monocytes increased following the injection of P2RX7A (Figures S5B-D). In all, our P2RX7A could alleviated monocyte-derived renal macrophage senescence, as well as the inflammatory response and kidney injury in septic AKI.

**Figure 6:**
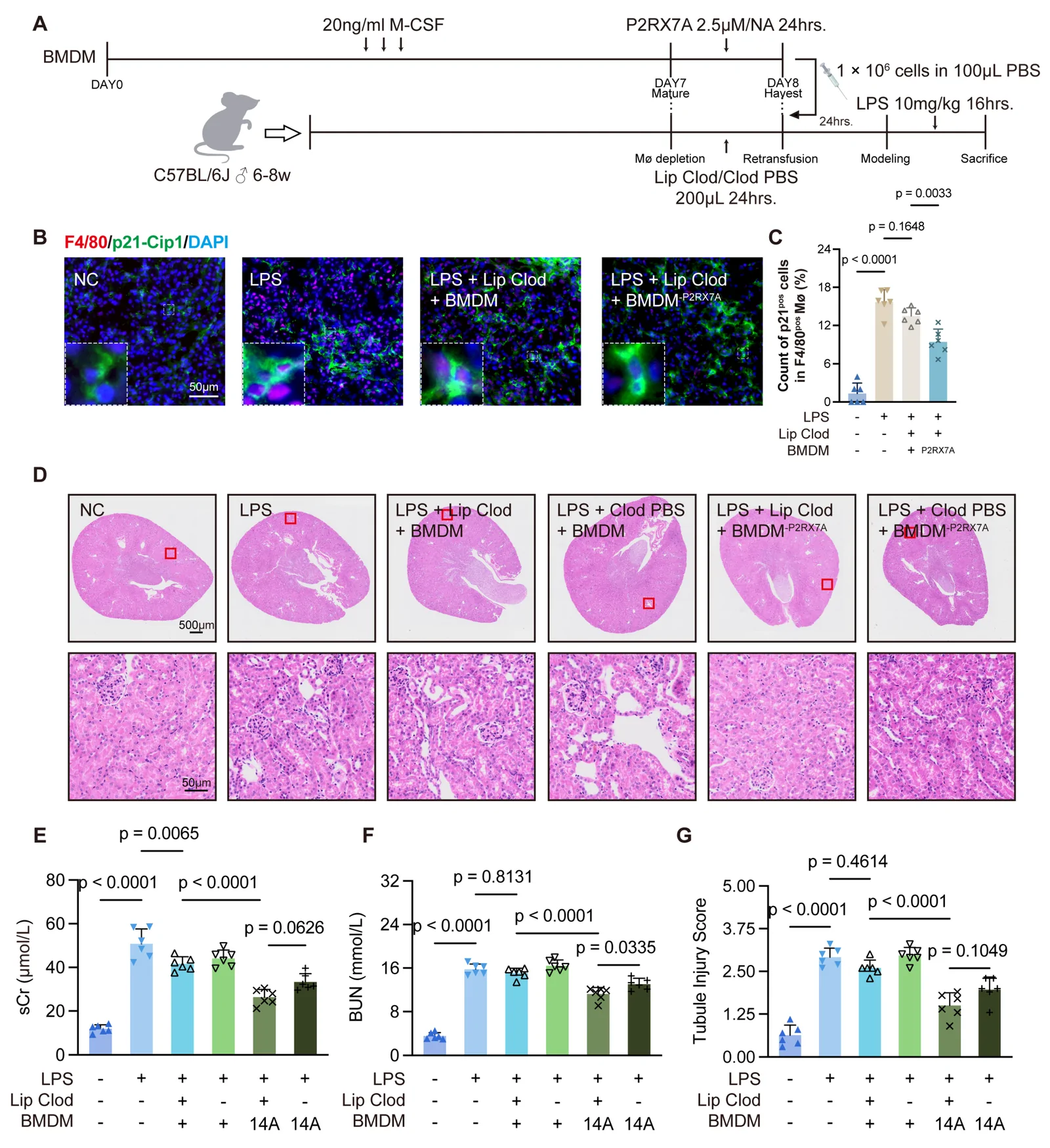
** Adoptive transfusion of BMDMs with P2RX7 antagonist pre-treatment alleviated kidney injury through targeting macrophage senescence in septic AKI.** (A) The schematic diagram of performing macrophage ablation and BMDM retransfusion pre-treated with P2RX7A on septic AKI mice. (B-C) The alteration in immunofluorescence of p21 in F4/80 identified macrophages in kidney sections after the retransfusion with BMDMs pre-treated with P2RX7A (200×). (D-G) The alteration in SCr and BUN level, tubule injury score and H&E staining (1× and 200×) of control and septic AKI mice after the retransfusion procedure with BMDMs pre-treated with P2RX7A (n = 6). Lipo Clod, clodronate liposomes.

Furthermore, to confirm whether it is P2RX7-associated renal macrophage senescence that participate in the tissue damage of septic AKI, we performed adoptively transplantation of BMDMs pre-treated with P2RX7A (BMDM^-P2RX7A^) into the mice before the LPS administration (Figure 6A). The reduced level of renal macrophage senescence was confirmed by the immunofluorescence co-staining F4/80 with p21 (Figures 6B-C), followed by a subsequent improvement in the kidney function and histological damage in the septic AKI mice (Figures 6D-G). We observe a slightly worse kidney function in septic AKI mice receiving BMDM^-P2RX7A^ transfusion in absence of the prior clodronate liposomes depletion. Therefore, we could conclude from the existed evidence that P2RX7A influenced the kidney injury in septic AKI through renal macrophage senescence.

To identify whether P2RX7A exerted its function on the vitro macrophage senescence, we stimulated BMDMs with the integration of LPS and ATP (Figure S6A). Such stimulation significantly induced the expression of cellular senescence signatures, the secretion of pro-inflammatory SASPs and the positive staining of senescence-associated beta-galactosidase (SA-β-gal), while P2RX7A effectively reversed these alterations (Figures S6B-K). High throughput RNA-seq figured out that almost a half of the genes in CellAge senescence signature database^30^ experiencing opposite differential expression across groups (Figure S6L). GSEA results revealed a decreased enrichment in the 'SASP' term after the administration of P2RX7A (Figure S6M). Similar results could be observed by the pharmacological antagonism and genetic inhibition of P2RX7 in THP-1 human leukemia monocytic cell line (Figures S7-8). Taken together, we further confirmed the role of P2RX7 and the efficacy of our small-molecule P2RX7A in LPS and ATP-stimulated macrophage senescence *in vitro*.

### P2RX7 antagonist rescued the dysfunction of senescent reparative macrophages for kidney repair

**Figure 7:**
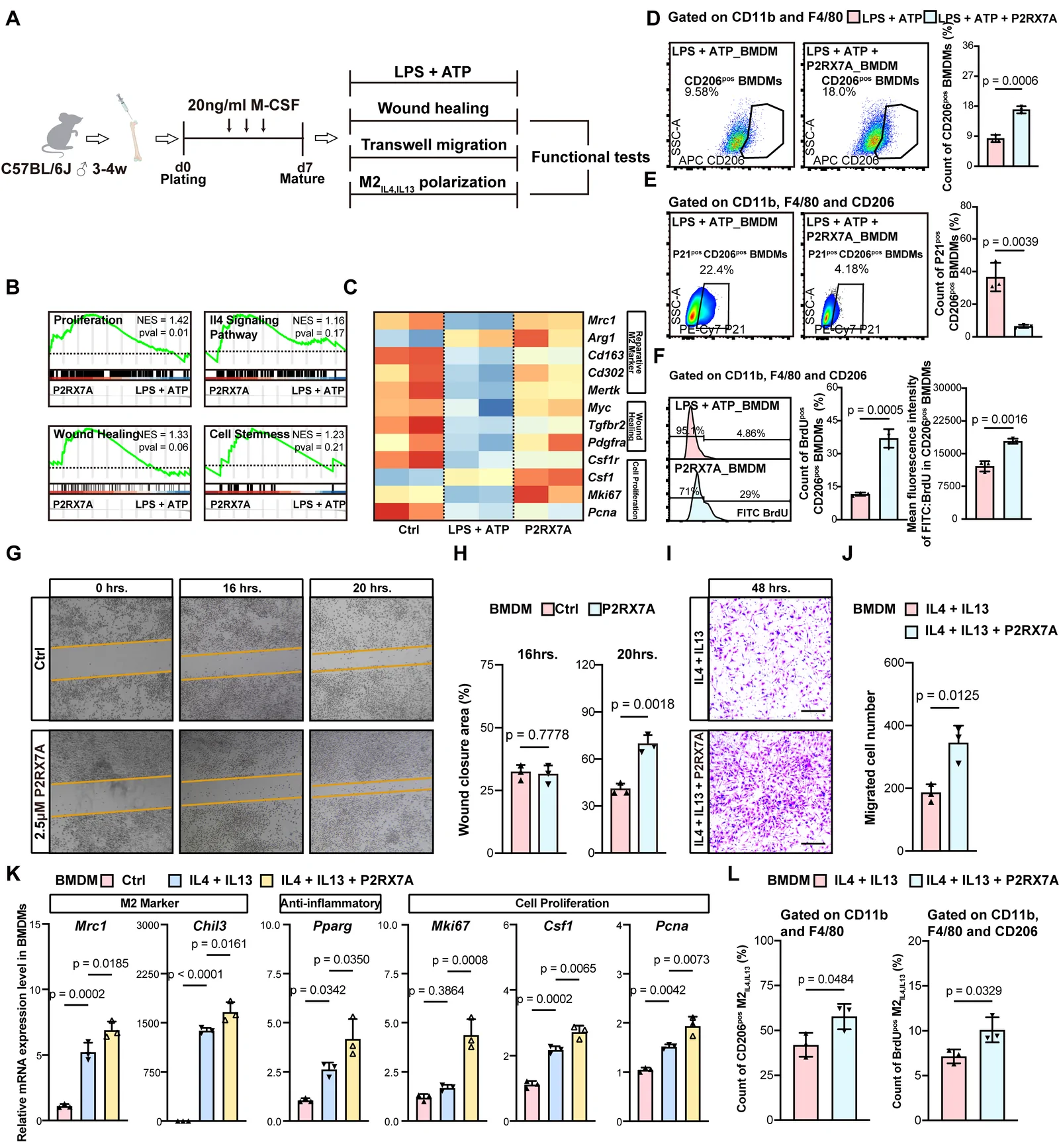
** P2RX7 antagonist could promote the capacity of senescent reparative macrophages in wound healing, migration and proliferation.** (A) Schematic diagram of extracting BMDMs and intervening BMDMs with P2RX7A in LPS and ATP co-stimulation model and several functional tests. (B) The heatmap of reparative, wound healing and proliferation marker genes in Ctrl, LPS + ATP, and LPS + ATP + P2RX7A group in high-array RNA sequencing. (C) GSEA enrichment result of the differential gene expression pattern changed in LPS + ATP + P2RX7A compared to LPS + ATP group, in up-regulation of the term 'proliferation', 'IL4 signaling pathway', 'wound healing' and 'cell stemness'. (D) The alteration in the count of CD206^pos^ CD11b^pos^ F4/80^pos^ BMDMs under the stimulation of LPS and ATP after the administration of P2RX7A (n = 3). (E-F) The alteration in the count of p21^pos^ and BrdU^pos^ CD206^pos^ CD11b^pos^ F4/80^pos^ BMDMs under the stimulation of LPS and ATP after the administration of P2RX7A (n = 3). (G-H) The alterations in the images and quantification of wound closure after 16hrs and 20hrs in the wound healing assay of BMDMs administrated with P2RX7A (n = 3, 40×). (I-J) The alterations in the images and quantification of transwell migration with the chemotaxis of IL4 and IL13 for 48hrs administrated of BMDMs with P2RX7A (n = 3, 100×); scale bar = 100μm. (K) The alterations in the mRNA level of reparative, anti-inflammatory and proliferation markers in IL4- and IL13-stimulated BMDMs administrated with P2RX7A (n = 3). (L) The alteration in the count of BrdU^pos^ CD206^pos^ CD11b^pos^ F4/80^pos^ BMDMs under the stimulation of IL4 and IL13 after the administration of P2RX7A (n = 3).

In order to figure out how P2RX7A attenuated the renal macrophage senescence in septic AKI, we reanalyzed the RNA-seq. An up-regulation in the expression of reparative macrophage markers, as well as genes related to 'cell proliferation', 'IL4 signaling pathway', 'wound healing' and 'cell stemness', accompanied with the decreased senescent state could be noticed in LPS- and ATP-stimulated BMDMs following the treatment of P2RX7A (Figures 7A-C). Using flow cytometry, we also observed an elevated count of CD206^pos^ CD11b^pos^ F4/80^pos^ reparative BMDMs in the P2RX7A treatment group (Figure 7D). Among these cells, the quantification of p21^pos^ CD206^pos^ senescent reparative BMDMs decreased, while BrdU^pos^ CD206^pos^ proliferative ones were restored (Figures 7E-F). In all, P2RX7A may alleviate the senescent condition of reparative macrophages through the recovery from their dysfunction in septic AKI.

We further confirmed our assumption through challenging BMDMs in some functional tests. In the wound healing assay, elevated efficiency of wound closure was found when P2RX7A was administrated (Figures 7G-H). Also, P2RX7A increased the number of migrating BMDMs in the transwell migration assay with the chemotaxis of IL4 and IL13 (Figures 7I-J). Furthermore, P2RX7A led to a more obvious pre-solving function in the IL4- and IL13-stimulated M2 macrophages (M2_IL4, IL13_), with the increased transcription of reparative macrophage markers *Mrc1* and *Chil3*, the anti-inflammatory marker *Pparg*, and proliferation markers *Mki67*, *Csf1* and *Pcna* (Figure 7K). Moreover, in P2RX7A-treated CD206^pos^ CD11b^pos^ F4/80^pos^ M2_IL4, IL13_, the count of BrdU^pos^ proliferative ones significantly elevated (Figure 7L). Taken together, these findings indicated that P2RX7A could further promote the capacity of M2_IL4, IL13_ in wound healing, migration and proliferation may account for the mechanism through which P2RX7A alleviated macrophage senescence in septic AKI.

### A long-acting, oral P2RX7 antagonist alleviated macrophage senescence and structural/functional changes of kidneys in aged mice

**Figure 8:**
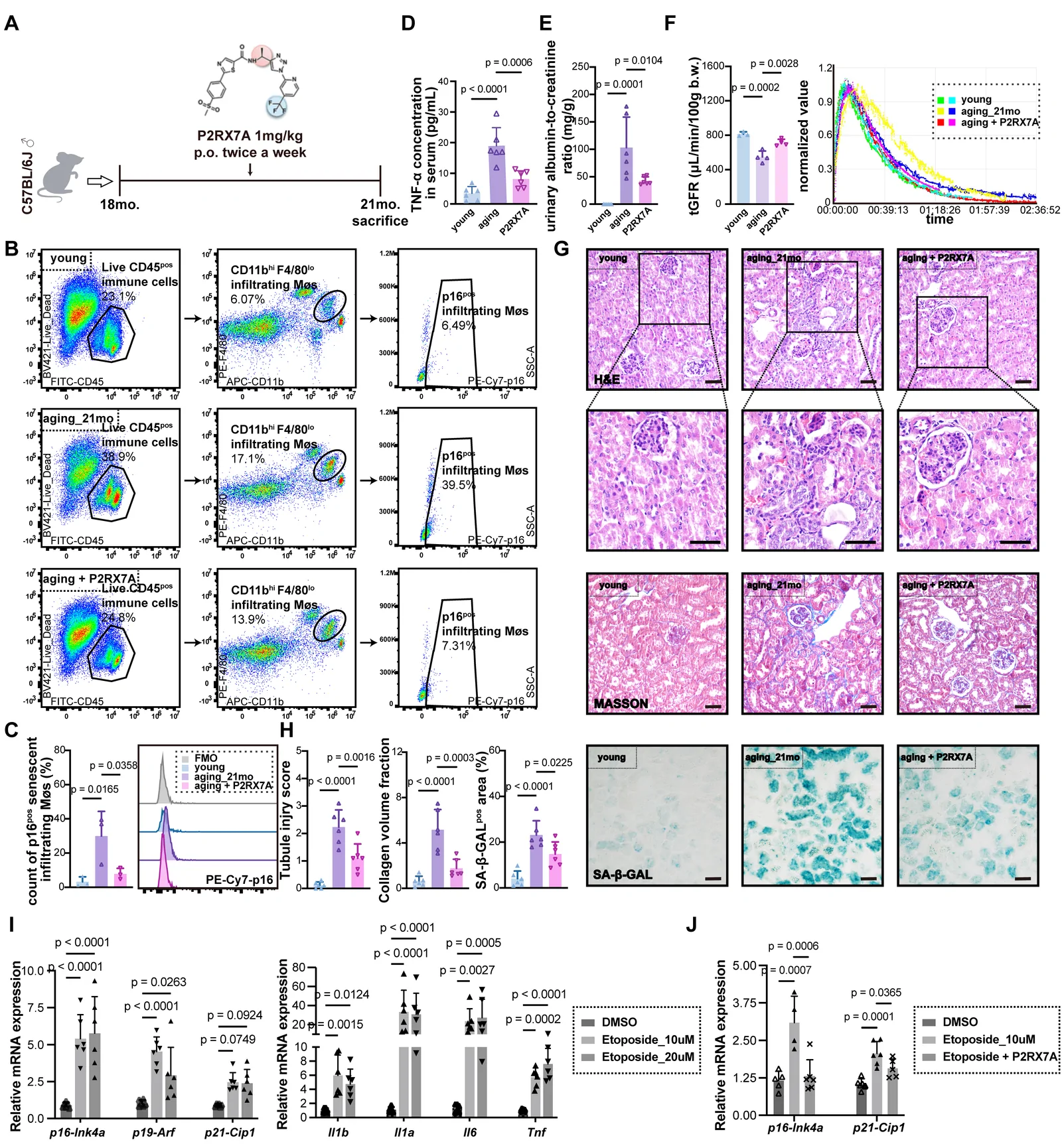
** A long-acting, oral, small-molecule P2RX7 antagonist delayed function decline, attenuated morphological changes and macrophage senescence in the kidney of aged mice.** (A) Schematic diagram of intervening aged mice with P2RX7A. (B-C) The quantification of p16^pos^ CD45^pos^ CD11b^hi^ F4/80^lo^ infiltrating macrophages in the kidneys of mice in each group (n = 3 per group). (D) Serum concentration of TNFα of mice in each group (n = 6 per group). (E) The quantification of urinary albumin-to-creatinine ratio of mice in each group (n = 6 per group). (F) The concentration time curves after i.v. injection of 7mg/100g FITC-Sinistrin into mice measured transcutaneously. The slope of the curves during the excretion period demonstrates the capacity of renal clearance of FITC-Sinistrin and suggests appropriateness of transcutaneous measurement for GFR determination. The transdermal glomerular filtration rates of mice in each group were calculated from the concentration time curves using MediBeacon Studio V2 software (n = 3-4 per group). (G-H) The H&E (200× and 400×), masson (200×) and senescence-associated beta-galactosidase (200×) staining, as well as the quantification of tubular injury score, collagen volume fraction and senescence-associated beta-galactosidase-positive area of mice in each group (n = 6 per group). Scale bar = 50µm. (I-J) The mRNA level of signatures concerning cell cycle arrest and senescence-associated secreted phenotypes in etoposide-induced senescent BMDMs changed with P2RX7A treatment (n = 5).

To clarify the roles of antagonizing P2RX7 in aged kidneys, we treated 18-month-old mice with 13A, a long-acting oral P2RX7A twice a week^22^ for three months (Figure 8A). First of all, we evaluated the therapeutic effects of P2RX7A on macrophage senescence during the aging process. We found that the quantification of p16^pos^ CD45^pos^ CD11b^hi^ F4/80^lo^ infiltrating macrophages was reduced in the kidney of aged mice treated with P2RX7A (Figures 8B-C). P2RX7A could alleviate systematic inflammation in aged mice (Figure 8D). As shown in Figures 8E-F, compared with their younger counterparts, old mice presented with reduced tGFR and increased urinary protein excretion, which were both relieved following the treatment of P2RX7A. Besides, old mice displayed evident tubular atrophy, tubule dilation, glomerulosclerosis and collagen deposition. The senescent state of the aged kidney could be confirmed by the remarkedly increased SA-β-gal-positive area. These morphological changes were all significantly alleviated in the P2RX7A group (Figures 8G-H). Taken together, the effect of targeting macrophage senescence against kidney injury through antagonizing P2RX7 could be further verified in the physiological aging model. Meanwhile, the anti-senescent effect of P2RX7A also could be observed on macrophages with cell cycle arrest induced by etoposide (Figures 8I-J).

## Discussion

Here, our study not only illustrates the crucial participation of senescent macrophages in septic AKI, but also puts forward our small-molecule P2RX7A as potential drugs against immunosenescence-associated kidney injury in sepsis and aging.

It has once been proposed that the onset of immunosenescence occurred earlier and acted as an important trigger of systematic organ aging^31^. Aged macrophages are challenged with the overall decreased capacity in pathogen phagocytosis, antigen-presenting and autophagy, constituting a significant part of immunosenescence. Though the gradual perception of premature senescence sheds lights on a new-type treatment for some intractable diseases, studies concentrating on the participation of immunosenescence in diseases are still limited. Tai *et al*. revealed the immunogenic features of cellular senescence that drived diabetic vascular injury, indicating a promising therapeutic strategy to improve vascular function in diabetes^12^. During the past time, we are inclined to focus on parenchymal cells when investigating kidney diseases since they are the first to bear the brunt. Our previous results have proposed that senescent proximal tubule cells were emerging as a therapeutic modality for AKI or CKD^1^,^4^. Functioning as a crucial driver both in the physiological and pathological scenarios, immune cells are complicated to interpret regarding their distinct characteristics in different stages of kidney diseases. Up till now, there is hardly any research exploring immunosenescence such as macrophage senescence in kidney injury.

Employing scRNA-seq data, our study is among the first to propose the possibility of targeting the senescent features of immune cells in kidney diseases and aging. In the injured kidneys of septic mice, macrophages were figured out to be the type of immune cells that underwent the most drastic alteration in their senescence gene signature expression pattern. Our results were in accordance with a recent study on rhabdomyolysis-associated AKI in which the authors observed an enrichment in cellular senescence among a certain group of macrophages^32^. Our results further extended the existing evidence through rigorous verification including the intravenous macrophage ablation and subsequent adoptive transfusion of BMDMs pre-treated with senolytics. Besides, considering there are both resident and infiltrating macrophages in the kidney, we also investigated the actual origin of senescent macrophages in septic AKI. Our results confirm that p21^hi^ CD45^pos^ renal immune cells were mainly constituted of F4/80^lo^ CD11b^hi^ infiltrating macrophages of myeloid origin. In the meantime, *C1Q^hi^* monocytes, which mediated pro-inflammatory effect in other autoimmune diseases^29^, displayed the most evident senescence signatures in the PBMCs of septic individuals. Therefore, our results reveal the potential of intervening senescent circulating mononuclear phagocytic cells against kidney diseases.

The phenotypic plasticity of macrophages is determined by the dynamic integration of multiple signaling pathways. Despite the general function decline of macrophages, how immunosenescence disturbs their specific phenotypes remains intricate. Based on the results of trajectory analysis, we discovered a certain group of macrophages co-expressing p21 and reparative markers CD206, Arg1 and Chil3 in the later stage of the kidney injury, guiding us to concentrate on the compromised function of senescent pre-solving macrophages exhibiting an alternatively activated phenotype. Such dysfunction could eventually contribute to tissue impairment and aging. Our results align with that of Zhou *et al*. who illustrated that the decline in type 2 cytokine signaling in macrophages was a significant age-associated feature, bringing about physical dysfunction^17^. Besides, Horiba *et al*. also reported that the number of CD206+ macrophages significantly decreased in the aged skin dermis and the imbalanced ratio across different macrophage phenotypes was positively correlated with the senescent state of the tissue^18^.

Senescent renal macrophages could ultimately contribute to tissue impairement in kidney injury and aging through the following two aspects^33^. First of all, the central pillar of replicative immunosenescence is 'inflamaging', an chronic inflammatory status attributable to pro-inflammatory SASPs secreted by senescent immune cells. Through paracrine signaling, these bioactive molecules propagates inflammation from a local niche to the entire tissue^34^. Besides, the function decline in senescent reparative macrophage phenotype, such as the impaired capacities in resolving inflammation, clearing cellular debris, facilitating angiogenesis, and promoting extracellular matrix reconstruction, also compromise the repair of damaged tissue in the onset of kidney disease and aging. In summary, our results reinforce previous research on the necessity of intervening senescent pre-solving macrophages, which are pivotal in orchestrating immunomodulation and promoting tissue repair.

Numerous types of stress from the outside are entangled in senescence, among which extracellular ATP (eATP) is emerging as a multifunctional metabolite with profound ramifications^35^. In kidney injuries, influenced by plentiful stimuli such as mechanical stress, inflammation and hypoxia, the injured tubular epithelial cells release excessive eATP^36^. Growing evidence pinpoints to the eATP-purinergic receptor (PR)-induced mitochondria impairment as well as its end degraded products as detrimental factors that drive cellular senescence^37^,^38^,^39^. For instance, the eATP-P2Y signaling has been testified to be an indispensable transducer linking external stress to intracellular senescence-associated alterations in hematopoietic stem cells and fibroblasts^36^,^37^. Similarly, our study found that P2RX7 was among the top in the whole PR family, experiencing a prominent and consistent up-regulation along time axis merely in macrophages. For the first time, conclusive evidence demonstrated the efficacy of disturbing P2RX7 in macrophages for alleviating kidney injury during septic AKI and aging. In addition, a co-expression pattern of P2RX7 and senescence gene signatures in mononuclear phagocytic cells facilitated us in unmasking the pivotal role of P2RX7 in the eATP-associated immunosenescence of kidney injury for the first time. Mechanistically, we indicated that P2RX7A could promote the wound healing, migration and proliferation capacity of senescent CD206^+^ pre-solving macrophages in kidney injury.

Taken together, these results expand our comprehension of the underlying mechanism regarding the immune microenvironment in injured kidney and offer novel insights into the plausibility of targeting macrophage senescence against kidney diseases. However, there are some limitations. First of all, AAMs also have some pathological phenotypes that have been confirmed to be profibrotic and contribute to AKI-to-CKD onset^42^. How immunosenescence affects the balance among these phenotypes warrants future exploration. Besides, the elderly are more suspicious to AKI. The effect of our small-molecule P2RX7A in the preventive and therapeutic effect against AKI of the aged population is worth exploring. In all, our discovered small-molecule P2RX7 antagonists may facilitate senescent renal reparative macrophages in the recovery from their dysfunction which has been compromised in kidney aging and diseases.

## Supplementary Material

Supplementary methods, figures and tables.

## Data Availability

The processed data of scRNA-seq of mouse renal cells obtained at multiple time points following LPS administration was downloaded from GEO (https://www.ncbi.nlm.nih.gov/, GSE151658). The raw count matrix of scRNA-seq of human peripheral blood mononuclear cells isolated from patients with sepsis was downloaded from Single Cell Portal (https://singlecell.broadinstitute.org/single_cell, SCP548). The raw and processed RNA-seq data has been deposited in GEO (GSE337932).

## References

[1] Yang L, Wang B, Guo F (2022). FFAR4 improves the senescence of tubular epithelial cells by AMPK/SirT3 signaling in acute kidney injury. Signal Transduct Target Ther.

[2] Wen L, Ren Q, Guo F (2023). Tubular aryl hydratocarbon receptor upregulates EZH2 to promote cellular senescence in cisplatin-induced acute kidney injury. Cell Death Dis.

[3] Li C, Xie N, Li Y (2019). N-acetylcysteine ameliorates cisplatin-induced renal senescence and renal interstitial fibrosis through sirtuin1 activation and p53 deacetylation. Free Radic Biol Med.

[4] Li L, Xiang T, Guo J (2024). Inhibition of ACSS2-mediated histone crotonylation alleviates kidney fibrosis via IL-1β-dependent macrophage activation and tubular cell senescence. Nat Commun.

[5] Xie H, Yang N, Lu L Uremic Toxin Receptor AhR Facilitates Renal Senescence and Fibrosis via Suppressing Mitochondrial Biogenesis. Adv Sci (Weinh). Published online June 28, 2024:e2402066.

[6] Wang W, Wu W (2025). Therapeutic Effects of Quercetin on Renal Fibrosis and Injury. Integr Med Nephrol Androl.

[7] Vicente R, Mausset-Bonnefont AL, Jorgensen C (2016). Cellular senescence impact on immune cell fate and function. Aging Cell.

[8] Liu Z, Liang Q, Ren Y (2023). Immunosenescence: molecular mechanisms and diseases. Signal Transduct Target Ther.

[9] Prieto LI, Sturmlechner I, Graves SI (2023). Senescent alveolar macrophages promote early-stage lung tumorigenesis. Cancer Cell.

[10] Haston S, Gonzalez-Gualda E, Morsli S (2023). Clearance of senescent macrophages ameliorates tumorigenesis in KRAS-driven lung cancer. Cancer Cell.

[11] Gulen MF, Samson N, Keller A (2023). cGAS-STING drives ageing-related inflammation and neurodegeneration. Nature.

[12] Tai GJ, Ma YJ, Feng JL (2025). NLRP3 inflammasome-mediated premature immunosenescence drives diabetic vascular aging dependent on the induction of perivascular adipose tissue dysfunction. Cardiovasc Res.

[13] Yao J, Li Y, Wang H (2023). The Roles of Myeloid Cells in Aging-related Liver Diseases. Int J Biol Sci.

[14] Kuwabara S, Goggins E, Okusa MD (2022). The Pathophysiology of Sepsis-Associated AKI. Clin J Am Soc Nephrol.

[15] Chen S, Saeed AFUH, Liu Q (2023). Macrophages in immunoregulation and therapeutics. Signal Transduct Target Ther.

[16] Lee S, Huen S, Nishio H (2011). Distinct macrophage phenotypes contribute to kidney injury and repair. J Am Soc Nephrol.

[17] Zhou Z, Yao J, Wu D (2024). Type 2 cytokine signaling in macrophages protects from cellular senescence and organismal aging. Immunity.

[18] Horiba S, Kami R, Tsutsui T (2022). IL-34 Downregulation-Associated M1/M2 Macrophage Imbalance Is Related to Inflammaging in Sun-Exposed Human Skin. JID Innovations.

[19] Qian Y, Qian C, Xie K (2021). P2X7 receptor signaling promotes inflammation in renal parenchymal cells suffering from ischemia-reperfusion injury. Cell Death Dis.

[20] Koo TY, Lee JG, Yan JJ (2017). The P2X7 receptor antagonist, oxidized adenosine triphosphate, ameliorates renal ischemia-reperfusion injury by expansion of regulatory T cells. Kidney International.

[21] Zhang R, Su K, Yang L (2023). Design, Synthesis, and Biological Evaluation of Novel P2X7 Receptor Antagonists for the Treatment of Septic Acute Kidney Injury. J Med Chem.

[22] Zhang R, Su K, Yang L (2024). Discovery of a Potent, Orally Active, and Long-Lasting P2X7 Receptor Antagonist as a Preclinical Candidate for Delaying the Progression of Chronic Kidney Disease. J Med Chem.

[23] Percie du Sert N, Hurst V, Ahluwalia A (2020). The ARRIVE guidelines 2.0: Updated guidelines for reporting animal research. Br J Pharmacol.

[24] Wang B, Xu J, Ren Q (2022). Fatty acid-binding protein 4 is a therapeutic target for septic acute kidney injury by regulating inflammatory response and cell apoptosis. Cell Death Dis.

[25] Janosevic D, Myslinski J, McCarthy TW (2021). The orchestrated cellular and molecular responses of the kidney to endotoxin define a precise sepsis timeline. Elife.

[26] Yao W, Chen Y, Li Z (2022). Single Cell RNA Sequencing Identifies a Unique Inflammatory Macrophage Subset as a Druggable Target for Alleviating Acute Kidney Injury. Adv Sci (Weinh).

[27] Huang W, Wang BO, Hou YF (2022). JAML promotes acute kidney injury mainly through a macrophage-dependent mechanism. JCI Insight.

[28] Reyes M, Filbin MR, Bhattacharyya RP (2020). An immune-cell signature of bacterial sepsis. Nat Med.

[29] Zheng W, Wang X, Liu J (2022). Single-cell analyses highlight the proinflammatory contribution of C1q-high monocytes to Behçet's disease. Proc Natl Acad Sci U S A.

[30] de Magalhães JP, Curado J, Church GM (2009). Meta-analysis of age-related gene expression profiles identifies common signatures of aging. Bioinformatics.

[31] Yousefzadeh MJ, Flores RR, Zhu Y (2021). An aged immune system drives senescence and ageing of solid organs. Nature.

[32] Rao SN, Zahm M, Casemayou A (2024). Single-cell RNA sequencing identifies senescence as therapeutic target in rhabdomyolysis-induced acute kidney injury. Nephrol Dial Transplant.

[33] Ai S, Zhu J, Wu X (2025). Macrophages: Novel Molecular Mechanism and Therapeutic Strategy in Diabetic Kidney Disease. Integrative Medicine in Nephrology and Andrology.

[34] Han Z, Wang K, Ding S, Zhang M (2024). Cross-talk of inflammation and cellular senescence: a new insight into the occurrence and progression of osteoarthritis. Bone Res.

[35] Pini M, Czibik G, Sawaki D (2021). Adipose tissue senescence is mediated by increased ATP content after a short-term high-fat diet exposure. Aging Cell.

[36] Solini A, Usuelli V, Fiorina P (2015). The dark side of extracellular ATP in kidney diseases. J Am Soc Nephrol.

[37] Guo Y, Guan T, Shafiq K (2023). Mitochondrial dysfunction in aging. Ageing Res Rev.

[38] Miwa S, Kashyap S, Chini E, von Zglinicki T (2022). Mitochondrial dysfunction in cell senescence and aging. J Clin Invest.

[39] Basisty N, Kale A, Jeon OH (2020). A proteomic atlas of senescence-associated secretomes for aging biomarker development. PLoS Biol.

[40] Volonte D, Benson CJ, Daugherty SL, Beckel JM, Trebak M, Galbiati F (2024). Purinergic signaling promotes premature senescence. J Biol Chem.

[41] Cho J, Yusuf R, Kook S (2014). Purinergic P2Y₁₄ receptor modulates stress-induced hematopoietic stem/progenitor cell senescence. J Clin Invest.

[42] Lan HY (2022). Macrophage-myofibroblast Transition in Kidney Disease. Integrative Medicine in Nephrology and Andrology.

