# Supplementary material for: Elimination of Senescent Macrophages by Purinergic Receptor P2RX7 Antagonist to Safeguard Against Kidney Injury and Aging
Source: Int J Biol Sci. 2026 Jul 30;22(13):7082–99. doi: 10.7150/ijbs.138299 (PMC13540451; doi:10.7150/ijbs.138299)
Supplement: Supplementary file 1 [file ijbsv22p7082s1.pdf]

SUPPLEMENTAL METHODS

Quantitative real-time PCR analysis

Total RNA was separated using Animal Total RNA Isolation Kit (RE-03014, FOREGENE, Chengdu, China), and reverse transcript cDNA using HiScript III RT SuperMix for qPCR (+gDNA wiper) (R323-01, Vazyme, Nanjing, China). The primers employed for target genes are presented in Supplementary Table 1. The PCR thermal cycling parameters were as follows: 95°C for 600s; 45 cycles of 95°C for 10s, 60°C for 15s and 72°C for 15s. The relative expression of genes in comparison with controls was normalized to the expression of GAPDH and calculated through CFX Manage Software (Bio-Rad, Hercules, CA, USA).

Western blot analysis

Total protein was extracted and separated by SDS-PAGE using 12% protein gels, and then transferred to PVDF membranes. The membranes were blocked with 5% nonfat milk at room temperature (RT) for 2hrs and then incubated with specific primary antibodies overnight at 4°C. The primary antibodies utilized are presented in Supplementary Table 2. After then, the membranes were incubated with appropriate secondary antibodies (HA1001/HA1006, Huabio, Hangzhou, China) for 2hrs at RT, and the bands were visualized with enhanced chemiluminescence reagent (BMU101, Abbkine, Wuhan, China). Immunoblots were observed through Odyssey infrared imaging system (Fluorescence Chemiluminescence Imaging System, Clinx Science, Shanghai, China) and quantified through utilizing ImageJ 6.0 software (Wayne Rasband, National Institutes of Health, USA). All of the western blotting assays were performed and repeated three times.

Hematoxylin and eosin (H&E) staining

One-quarter of the kidney was excised and then fixed in 10% formaldehyde (50-00-0, Chron Chemicals, Chengdu, China), dehydrated, embedded into the paraffin. Paraffin was sectioned at a thickness of 4μm for H&E staining. Images were gathered at 200× and 400× magnification under AxioCamHRc digital camera (Carl Zeiss, Jena, Germany) with ZEN 2012 software. Renal tubular injury was assessed on a semiquantitative scale of 0-4 as described below: 0, normal; 1, <25% injury; 2, 25-50% injury; 3, 51-75% injury; and 4, >75% injury. All assessments were done by two investigators blinded to the experimental settings.

Immunofluorescence

For animal experiments, OCT compounds embedding one-quarter of the kidney were sectioned at a thickness of 4μm and subsequently blocked at RT utilizing 10% 10 × horse serum (ZLI-9024, Beijing, China) for 1hr.

Cells were harvested and fixed using 4% PFA for 15min, then permeabilized and blocked using 0.1% Triton X-100 (T8200, SolarBio, Beijing, China) and 10% FBS for 1hr.

Next, tissue and cells were incubated with specific primary antibodies at 4°C for at least 16hrs and Alexa Fluor® 488 or TRITC-conjugated secondary antibodies (Jackson ImmunoResearch, West Grove, PA, USA) at RT for 2hrs. Tissue and cells were re-cleaned, stained with DAPI (D8200, Solarbio, Beijing, China), and sealed through coverslips. Images were gathered at 200× magnification under ECLIPSE Ts2R-FL research microscopy.

Senescence-associated β-galactosidase staining

In accordance with the guidelines of the Senescence β-Galactosidase Staining Kit (C0602, Beyotime Biotechnology, Shanghai, China), harvested cells were fixed for 15min, permeabilized for 30min and

stained in 37°C and low CO_2 incubator. Images were gathered at 100× under ECLIPSE Ts2R-FL (Nikon Instruments, Melville, NY, USA) utilizing the NIS-Elements D 5.21.00 software.

Small interfering RNA (siRNA) transfection

The human P2RX7 siRNA was purchased from GenePharma (Shanghai, China). THP-1 cells transfection with siRNAs was conducted using Lipofectamine 3000 (L3000075, Invitrogen, CA, USA) according to the manufacturer's instructions. The sequences were listed as follows: P2RX7 siRNA, sense 5′-CCUGUGUGGUCAACGAAUATT-3, and antisense 5′-UAUUCGUUGACCACACAGGTT-3; NC siRNA sense 5'-UUCUCCGAACGUGUCACGUTT-3', and antisense 5'-ACGUGACACGUUCGGAGAATT-3'

Mouse kidney function measurement

Blood samples were drawn from mouse eye socket and then centrifuged at 3000 rpm for 20min at 4°C to separate serum. The levels of serum urea and creatinine, as well as urine microalbumin and creatinine were determined with an automated biochemical analyzer (Mindray BS-240, Shenzhen, China).

RNA-sequencing

Total RNA of stimulated-BMDMs cultured in 10mm petri dishes (n = 3 per group) were extracted using TRIzol reagent (Invitrogen, Carlsbad, CA, USA) and sent to LC-BIO Bio-Tech Ltd (Hangzhou, China) for sequencing. Samples first underwent purity, quality, and integrity test. With Illumina NovaSeq 6000 platform, constructed libraries were subsequently sequenced and paired-end reads with a 2 × 150 bp read length were produced.

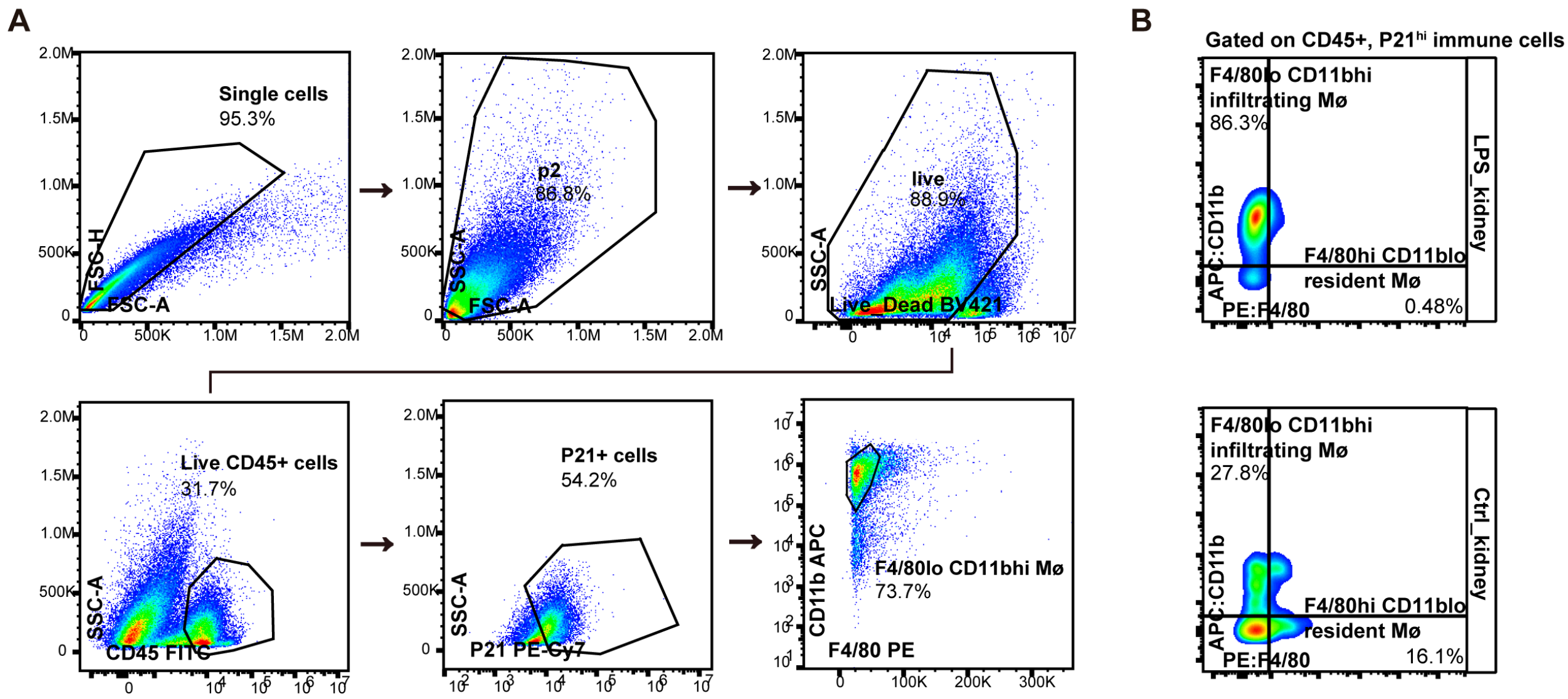

Figure S1 (related to Figure 1): (A) The gating strategy of flow cytometry measuring p21 expression in CD45^{pos} CD11b^{pos} F4/80^{pos} renal macrophages. (B) Quantification of p21^{pos} CD45^{pos} CD11b^{pos} F4/80^{pos} renal macrophages under healthy or septic AKI conditions.

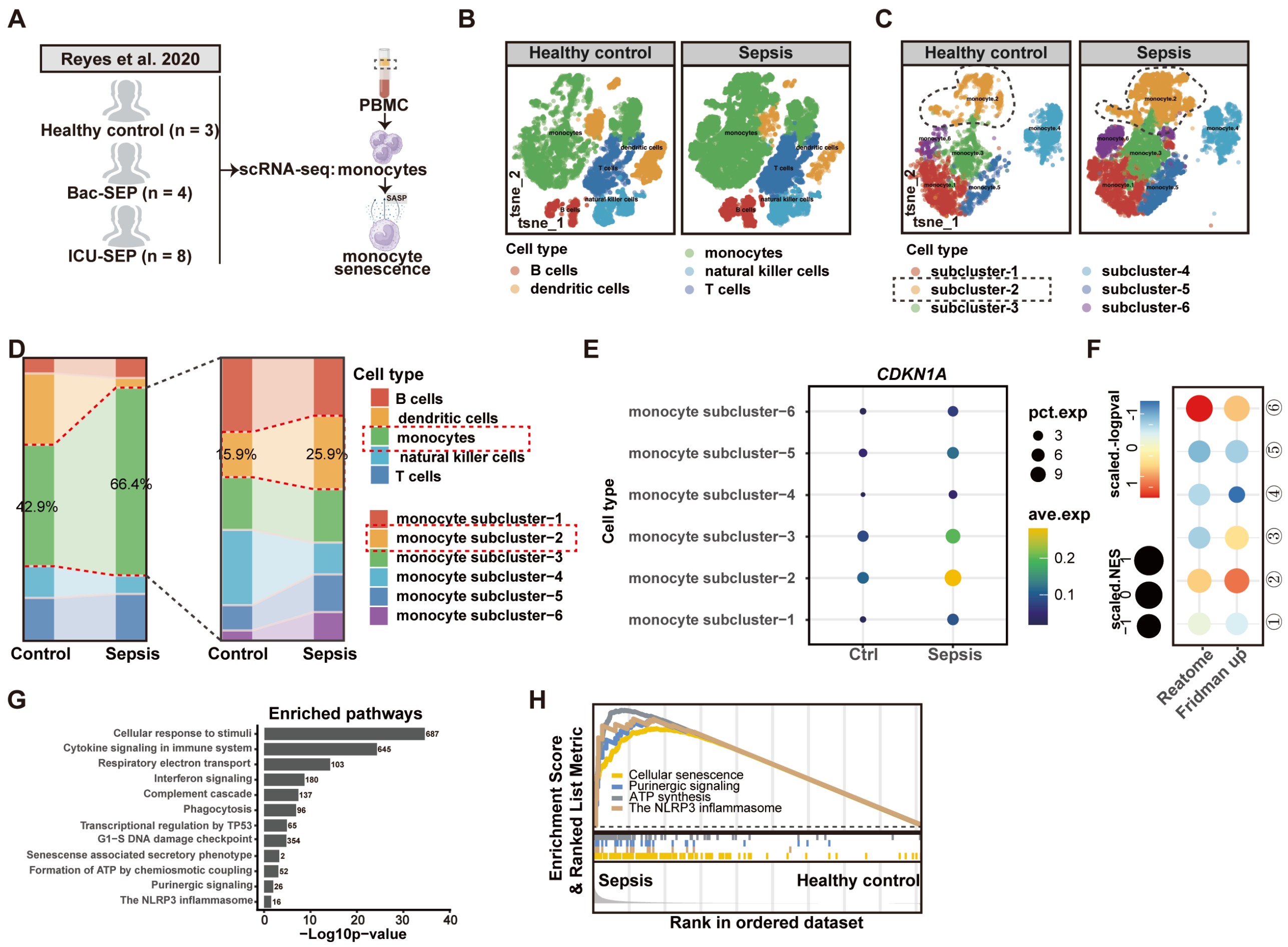

Figure S2 (A) The following analysis utilized single-cell transcriptomic data performed in healthy control and sepsis patients by Reyes et al. 2020 (Single Cell Portal SCP548). (B) The tSNE embedding of single cells from human peripheral blood mononuclear cells under healthy and septic conditions, colored by cluster identity. (C) The tSNE embedding of monocyte single cells under healthy and septic conditions, colored by cluster identity. (D) The proportion of major cell types as well as monocyte subtypes changed with the development of sepsis in the peripheral blood of patients. (E) Quantification of *CDKN1A* in blood monocyte subclusters under healthy and septic conditions. (F) GSEA enrichment results of the differential senescence gene expression patterns in monocyte subcluster-2 (*C1Q*^{hi} monocytes) changed in sepsis compared to healthy control. (G-H) GSEA enrichment results of the differential gene expression patterns in monocyte subcluster-2 (*C1Q*^{hi} monocytes) changed in septic patients, especially in ‘cellular senescence’, ‘purinergic signaling’, ‘ATP synthesis’ and ‘NLRP3 inflammasome’.

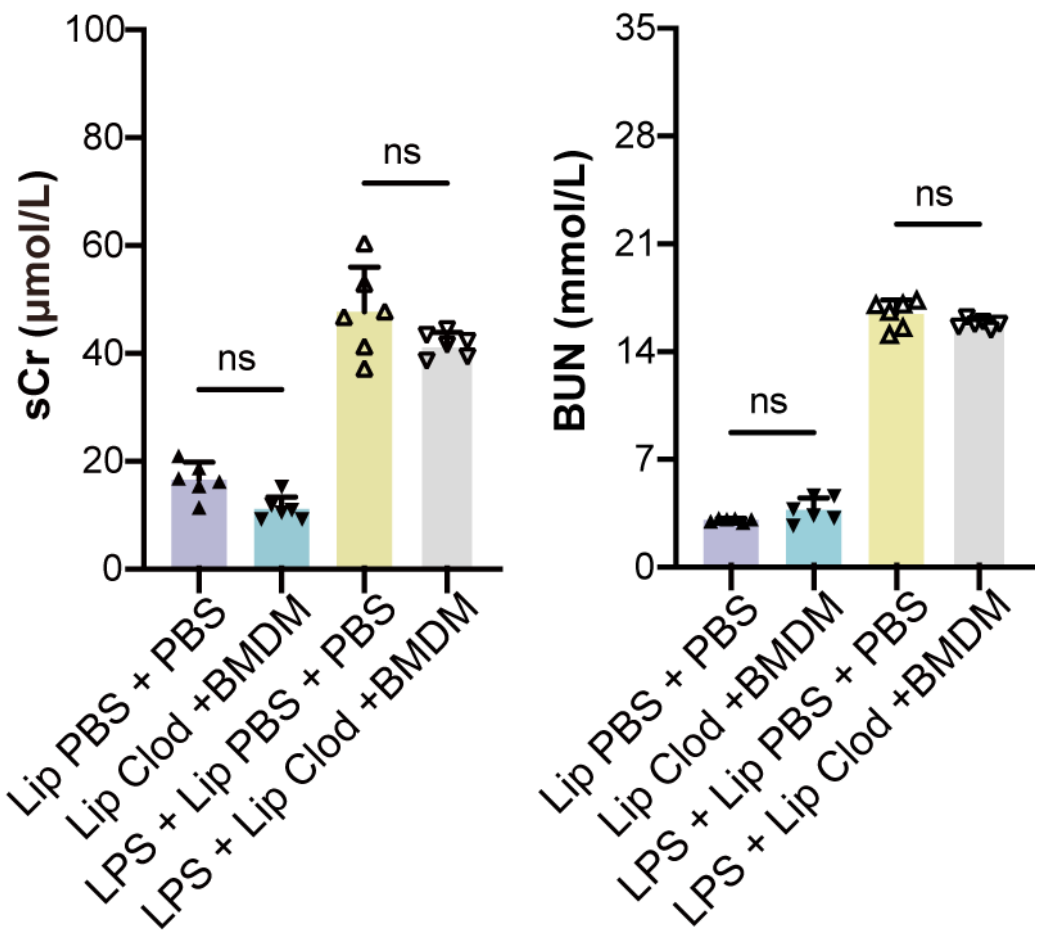

Figure S3 (related to Figure 2): The alteration in SCr and BUN level of control and septic AKI mice after the retransfusion procedure (n = 6).

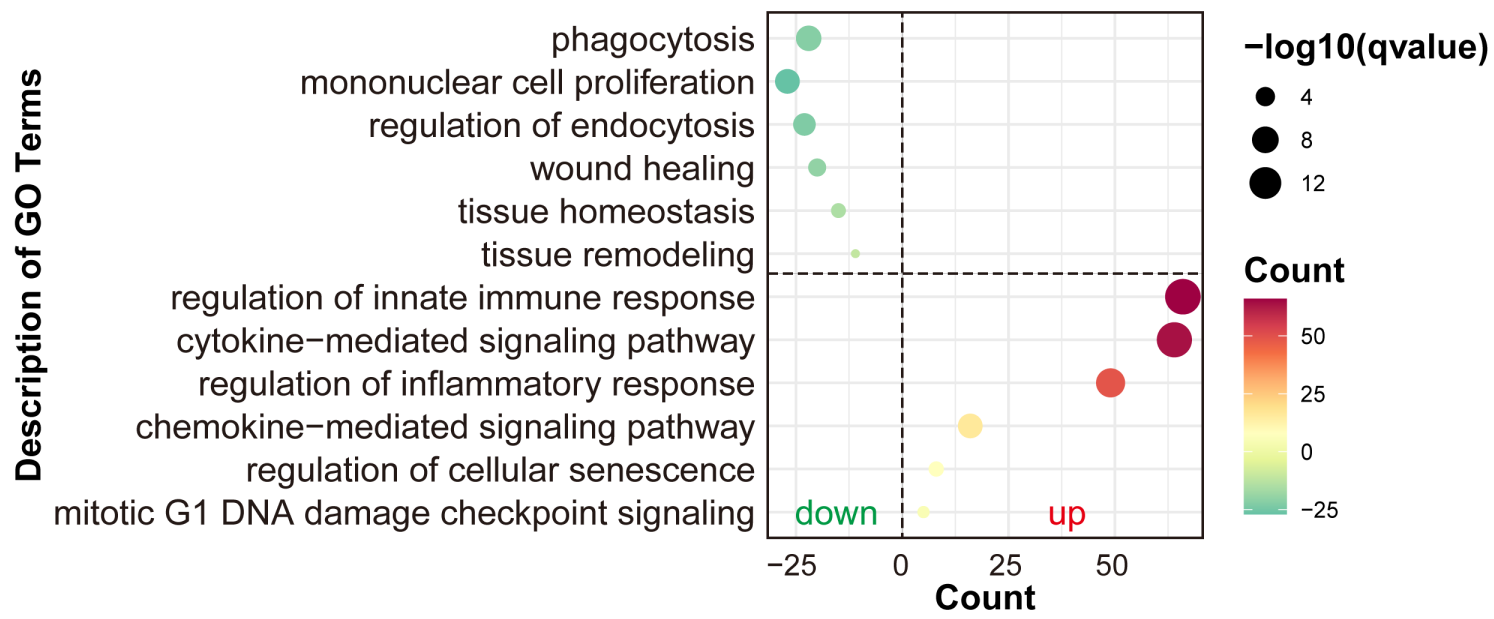

Figure S4 (related to Figure 3): The GO enrichment results of *Cdkn1a*^{pos} senescent renal macrophages in septic AKI mice compared with *Cdkn1a*^{neg} ones.

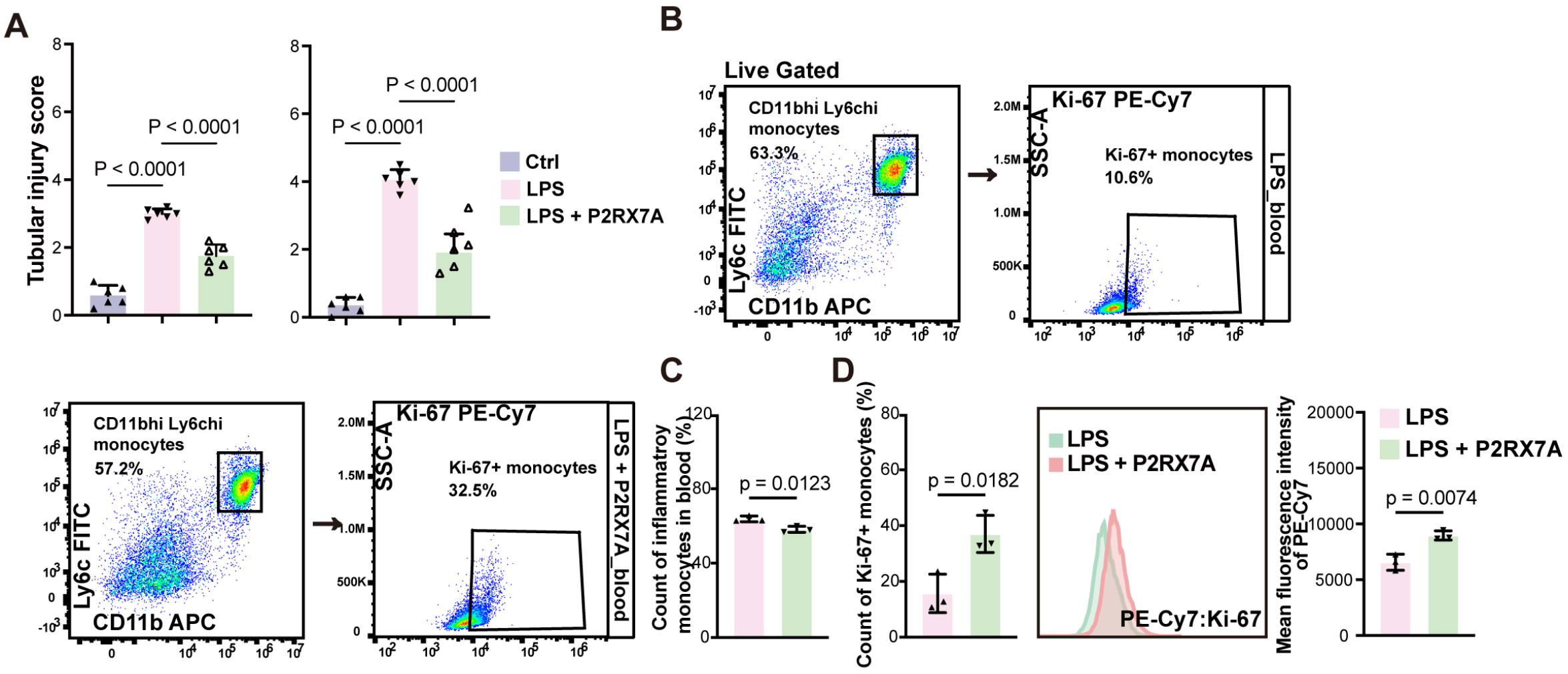

Figure S5 (related to Figure 5): (A) The alteration of tubule injury score of septic AKI mice after the administration of P2RX7A (n = 6). (B) The alteration in the count of $CD11b^{pos}$ $Ly6C^{pos}$ monocytes in the peripheral blood of septic AKI mice after the administration of P2RX7A (n = 3). (C-D) The alteration in the quantification of $Ki\text{-}67^{pos}$ $CD11b^{pos}$ $Ly6C^{pos}$ monocytes in the peripheral blood of septic AKI mice after the administration of P2RX7A (n = 3).

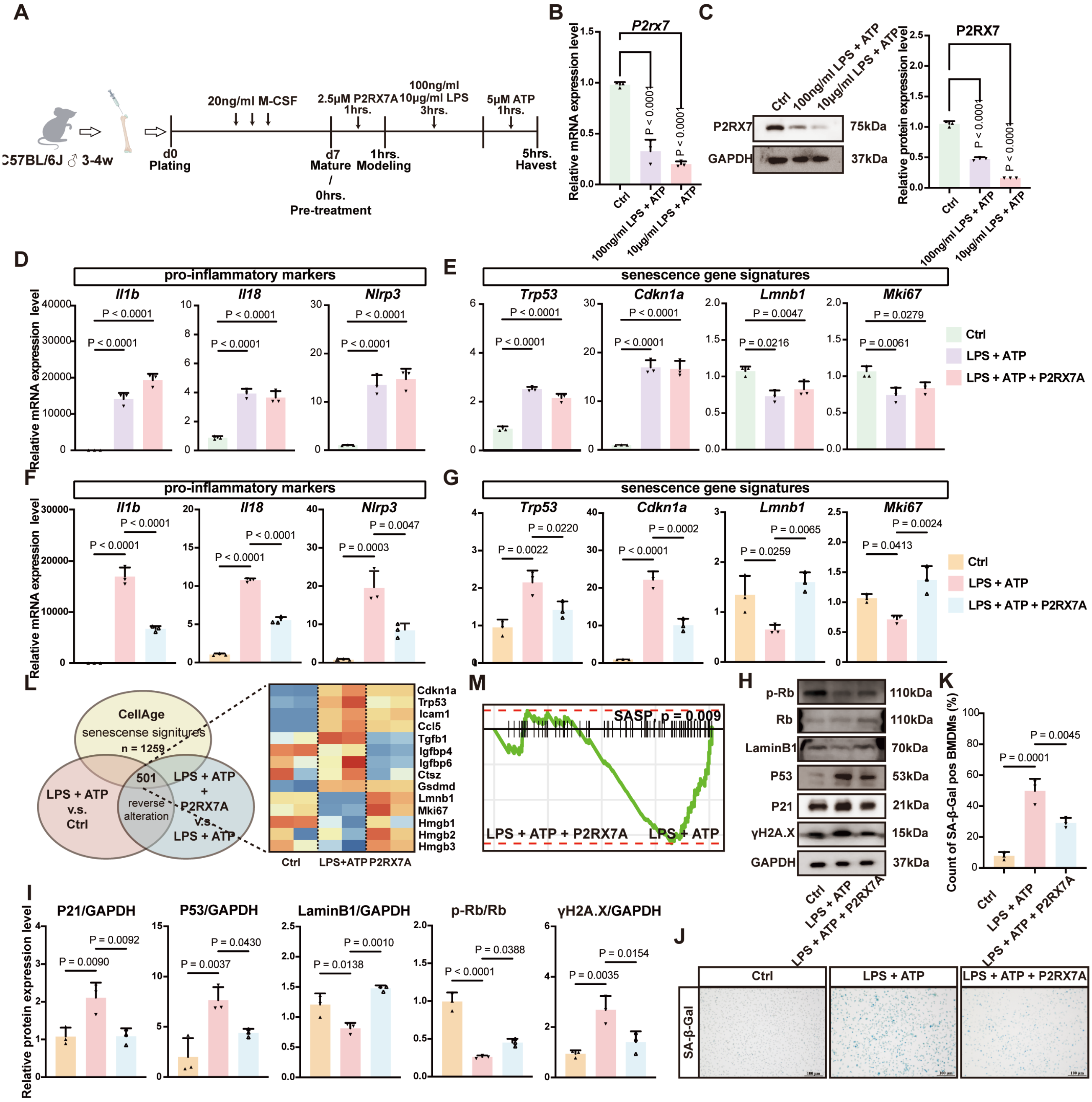

Figure S6 P2RX7A alleviated inflammation and cellular senescence in LPS/ATP-stimulated BMDMs. (A) Schematic diagram of extracting BMDMs and intervening LPS-stimulated BMDMs with P2RX7A. (B, C) The mRNA and protein level of P2RX7 in BMDMs changed with LPS stimulation of different doses (n = 3). (D, E) The mRNA level of inflammatory markers IL-1β, IL-18 and NLRP3 and senescence gene signatures p21, p53, Lamin B1, Ki-67 in BMDMs changed with LPS stimulation of different doses (n = 3). (F-I) The mRNA or protein level of inflammatory markers IL-1β, IL-18 and NLRP3 and senescence gene signatures p21, p53, Lamin B1, Ki-67, p-Rb/Rb and γH2A.X in BMDMs changed with P2RX7A treatment (n = 3). (J, K) The senescence-associated beta-galactosidase staining in BMDMs changed with P2RX7A treatment (n = 3). (L) Venn diagram and heatmap of CellAge senescence gene signatures experiencing reverse alteration in differential analysis of LPS + ATP *vs.* Ctrl and LPS + ATP + P2RX7A *vs.* LPS + ATP in high-array RNA sequencing. (M) GSEA enrichment result of the differential gene expression pattern changed in LPS + ATP +

P2RX7A compared to LPS + ATP group, in down-regulation of the term ‘senescence-associated secretory phenotypes’.

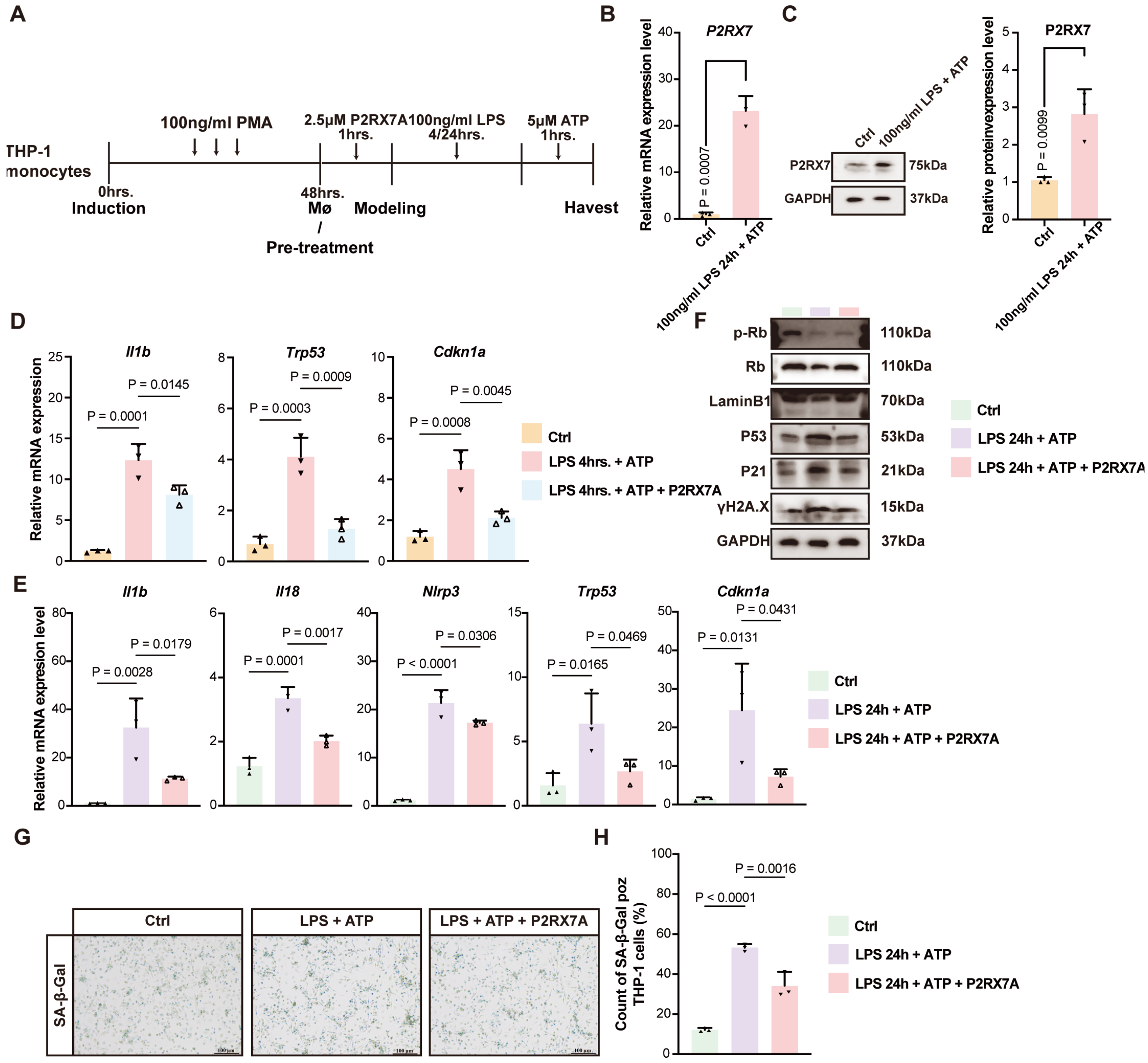

Figure S7 P2RX7A alleviated inflammation and cellular senescence in LPS/ATP-stimulated human monocytic THP-1 cells. (A) Schematic diagram of intervening LPS-stimulated THP-1 cells with P2RX7A. (B, C) The mRNA and protein level of P2RX7 in THP-1 cells changed with LPS stimulation (n = 3). (D) The mRNA level of inflammatory markers IL-1β and senescence gene signatures p21 and p53 in THP-1 cells changed with P2RX7A treatment under the stimulation of LPS for 4hrs (n = 3). (E, F) The mRNA or protein level of inflammatory markers IL-1β, IL-18 and NLRP3 and senescence gene signatures p21, p53, Lamin B1, Ki-67, p-Rb/Rb and γH2A.X in THP-1 cells changed with P2RX7A treatment under the stimulation of LPS for 24hrs (n = 3). (G, H) The senescence-associated beta-galactosidase staining in THP-1 cells changed with P2RX7A treatment (n = 3).

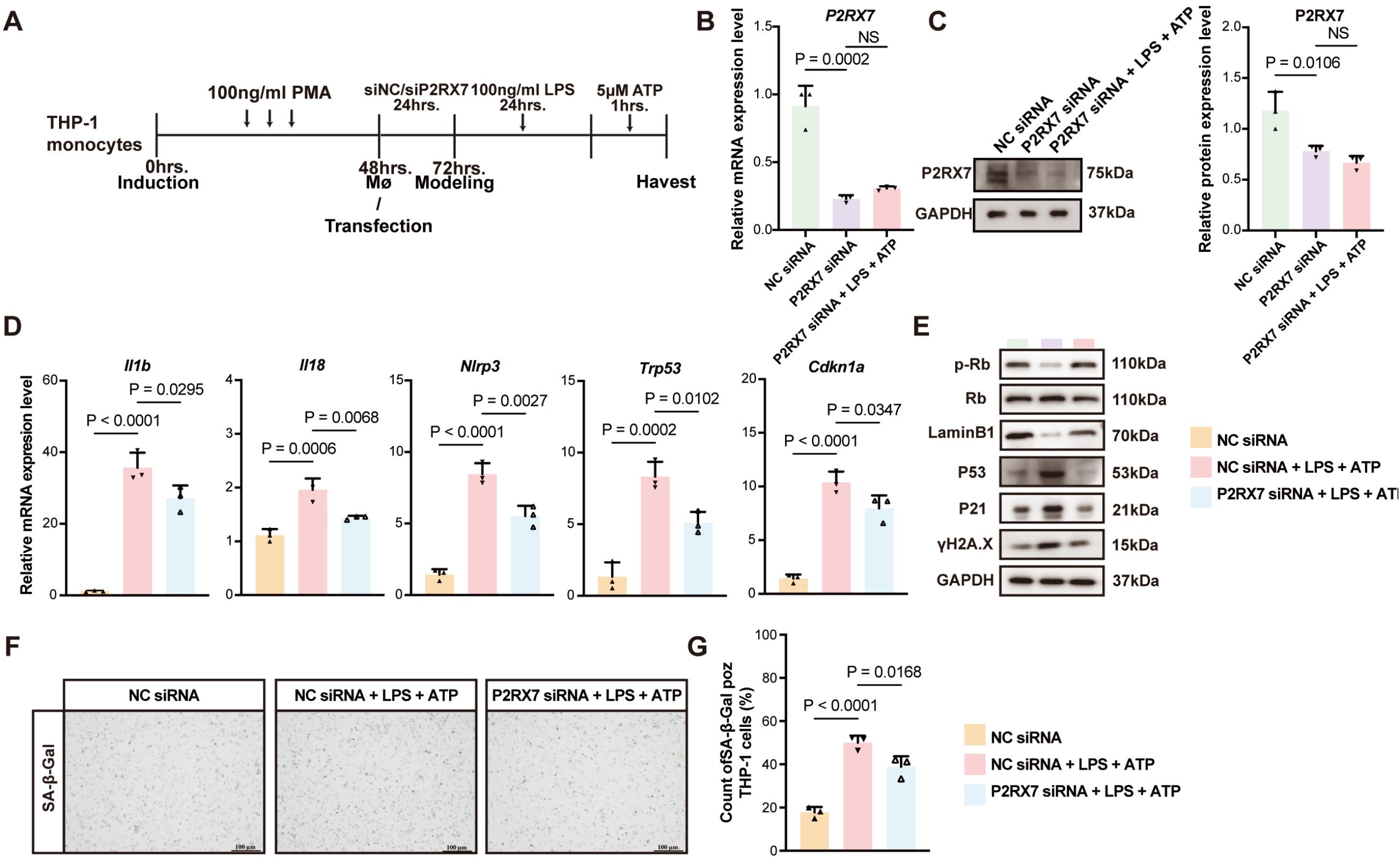

Figure S8 Genetically knockdown of P2RX7 alleviated cellular senescence in LPS/ATP-stimulated THP-1 cells. (A) Schematic diagram of intervening LPS-stimulated THP-1 cells with P2RX7 siRNA. (B, C) The mRNA and protein level of P2RX7 in THP-1 cells changed with P2RX7 siRNA treatment (n = 3). (D, E) The mRNA and protein level of inflammatory markers IL-1β, IL-18 and NLRP3 and senescence gene signatures p21, p53, Lamin B1, Ki-67, p-Rb/Rb and γH2A.X in THP-1 cells changed with P2RX7 siRNA treatment (n = 3). (F, G) The senescence-associated beta-galactosidase staining in THP-1 cells changed with P2RX7 siRNA treatment (n = 3).

Table S1 The list of primer sequences in the current study

gene symbol	primer sequence
mouse-P2rx7-f	5'-GACAAACAAAGTCACCCGGAT-3'
mouse-P2rx7-r	5'-CGCTCACCAAAGCAAAGCTAAT-3'
human-P2rx7-f	5'-TATGAGACGAACAAAGTCACTCG-3'
human-P2rx7-r	5'-GCAAAGCAAACGTAGGAAAAGAT-3'
mouse-Il1b-f	5'-TGGGCCTCAAAGGAAAGAAT-3'
mouse-Il1b-r	5'-CAGGCTTGTGCTCTGCTTGT-3'
human-Il1b-f	5'-ATGATGGCTTATTACAGTGGCAA-3'
human-Il1b-r	5'-GTCGGAGATTCGTAGCTGGA-3'
mouse-Il18-f	5'-CCTACTTCAGCATCCTCTACTGG-3'
mouse-Il18-r	5'-AGGGTTTCTTGAGAAGGGGAC-3'
human-Il18-f	5'-TCGGGAAGAGGAAAGGAACC-3'
human-Il18-r	5'-TTCTACTGGTTCAGCAGCCA-3'
mouse-Nlrp3-f	5'-ATTACCCGCCCGAGAAAGG-3'
mouse-Nlrp3-r	5'-TCGCAGCAAAGATCCACACAG-3'
human-Nlrp3-f	5'-AACATGCCCAAGGAGGAAGA-3'
human-Nlrp3-r	5'-GGCTGTTCACCAATCCATGA-3'
mouse-Trp53-f	5'-CCCCTGTCATCTTTTGTCCCT-3'
mouse-Trp53-r	5'-AGCTGGCAGAATAGCTTATTGAG-3'
human-Trp53-f	5'-GCCATCTACAAGCAGTCACAG-3'
human-Trp53-r	5'-TCATCCAAATACTCCACACGC-3'
mouse-Cdkn1a-f	5'-CCTGGTGATGTCCGACCTG-3'

mouse-Cdkn1a-r	5’-CCATGAGCGCATCGCAATC-3’
human-Cdkn1a-f	5’-TGTCACTGTCTTGTACCCTTG-3’
human-Cdkn1a-r	5’-GGCGTTTGGAGTGGTAGAA-3’
mouse-Lmnb1-f	5’-CCGGCCTCAAGGCTCTCTA-3’
mouse-Lmnb1-r	5’-TGCCGCCTCATACTCTCGAA-3’
mouse-Mki67-f	5’-ATCATTGACCGCTCCTTTAGGT-3’
mouse-Mki67-r	5’-GCTCGCCTTGATGGTTCCT-3’
mouse-Gapdh-f	5’-CCAATGTGTCCGTCGTGGATCT-3’
mouse-Gapdh-r	5’-GTTGAAGTCGCAGGAGACAACC-3’
Human-Gapdh-f	5’- ACAACTTTGGTATCGTGGAAGG -3’
human-Gapdh-r	5’- GCCATCACGCCACAGTTTC-3’

Table S2 The list of primary antibodies in the current study

**name**	**company**	**catalog Number**	**application**
anti-p53 [C8-A11]	HUABIO	M1312-2	WB
anti-p21 Cip1	HUABIO	HA722065	WB, IF，FC
anti-p16 INK4A	Abcam	ab211542	FC
anti-Ki67 [SR00-02]	HUABIO	HA721115	FC
anti-phospho-Histone H2A.X (Ser139)	Cell Signaling Technology<br>HUABIO	2577<br>ET1602-2	WB, IF
anti-Rb (D20)	Cell Signaling Technology	9313	WB
anti-phospho-Rb (Ser807/811) (D20B12)	Cell Signaling Technology	8516	WB
anti-LaminB1 (D9V6H)	Cell Signaling Technology	13435	WB
anti-GAPDH	Zen-Bioscience	200306-7E4	WB

Unaltered/Unedited Images

Figure S6C

P2RX7 (75 kDa)

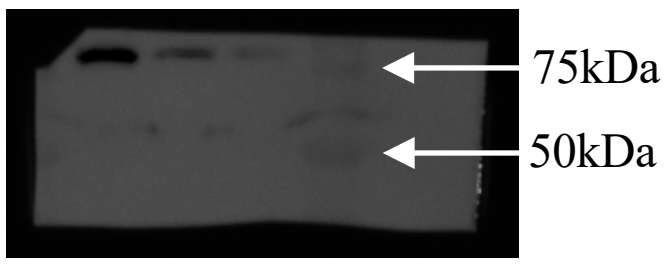

GAPDH (37 kDa)

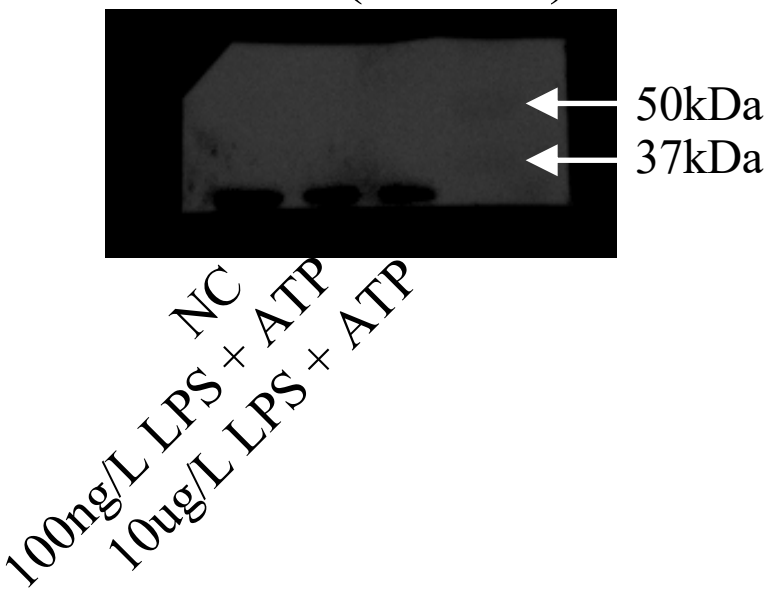

Figure S6H

p-Rb (110 kDa)

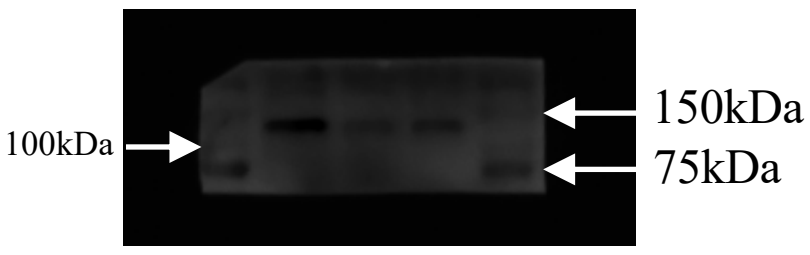

Rb (110 kDa)

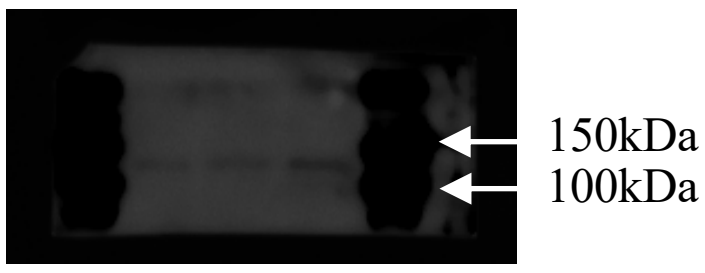

Lamin B1 (70 kDa)

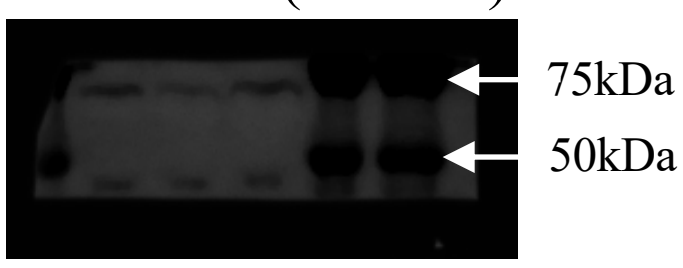

p53 (53 kDa)

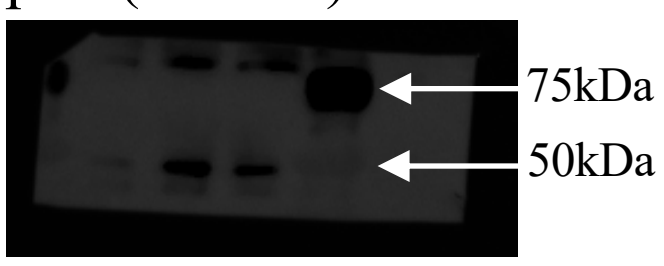

GAPDH (37 kDa)

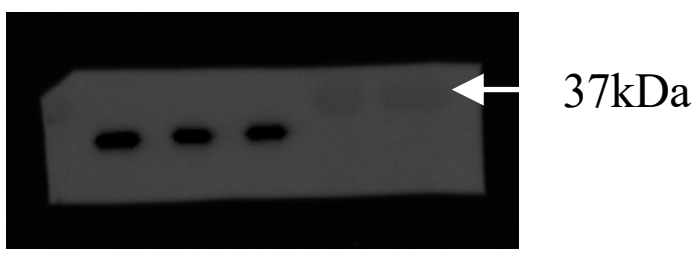

p21 (21 kDa)

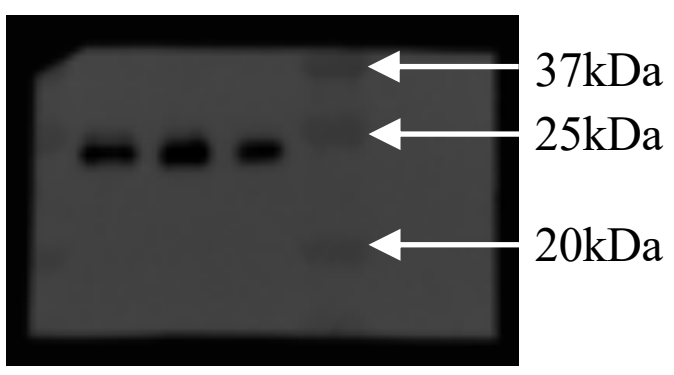

yH2A.X (15 kDa)

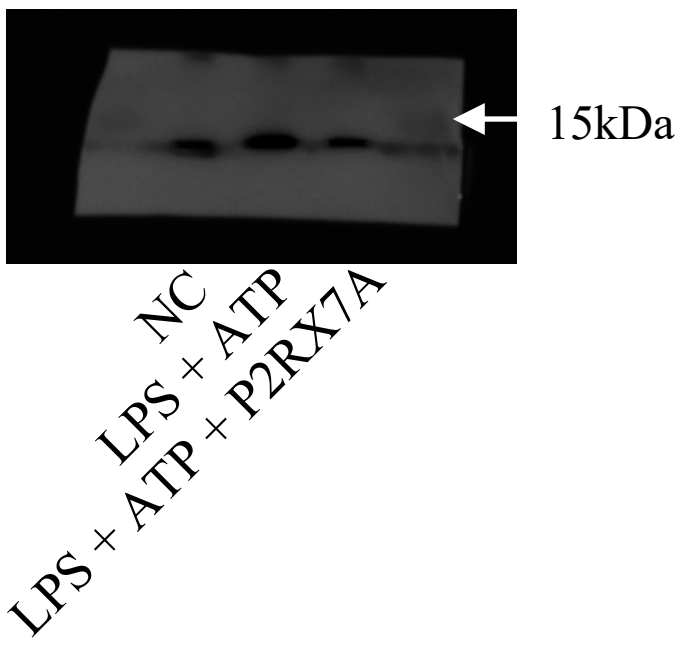

Figure S7C

P2RX7 (75 kDa)

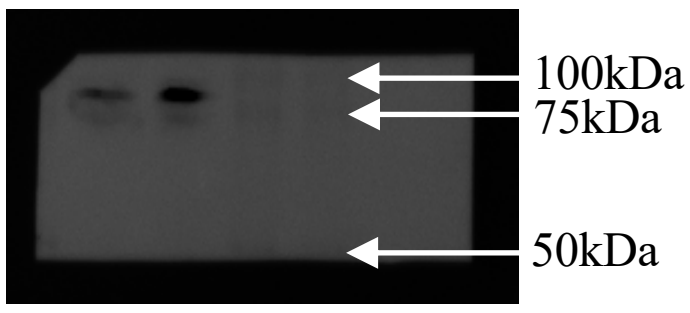

GAPDH (37 kDa)

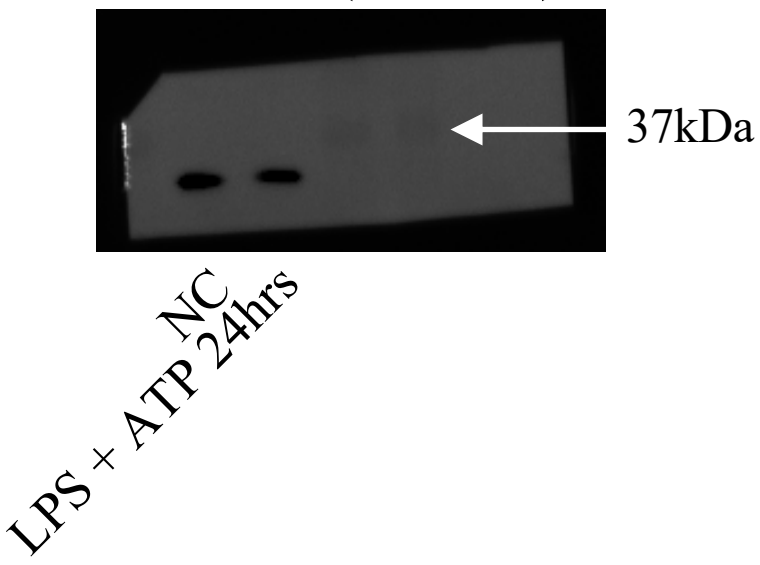

Figure S7F

p-Rb (110 kDa)

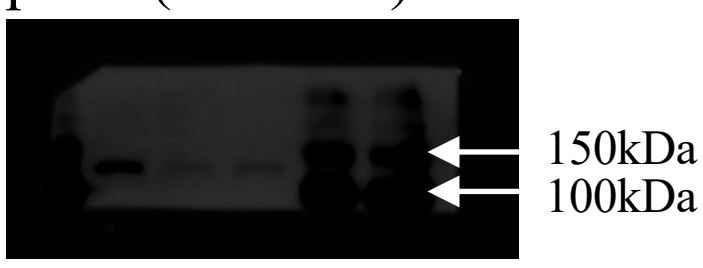

Rb (110 kDa)

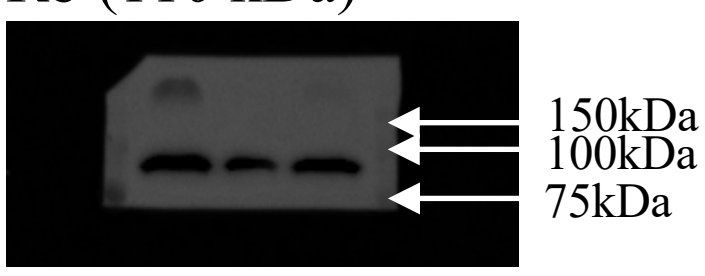

Lamin B1 (70 kDa)

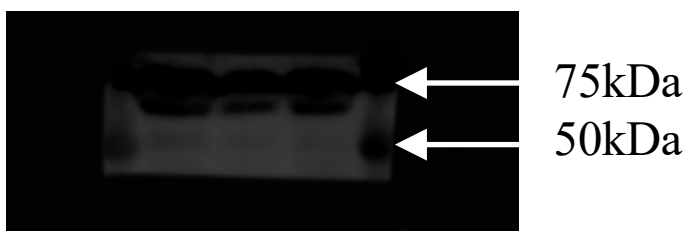

p53 (53 kDa)

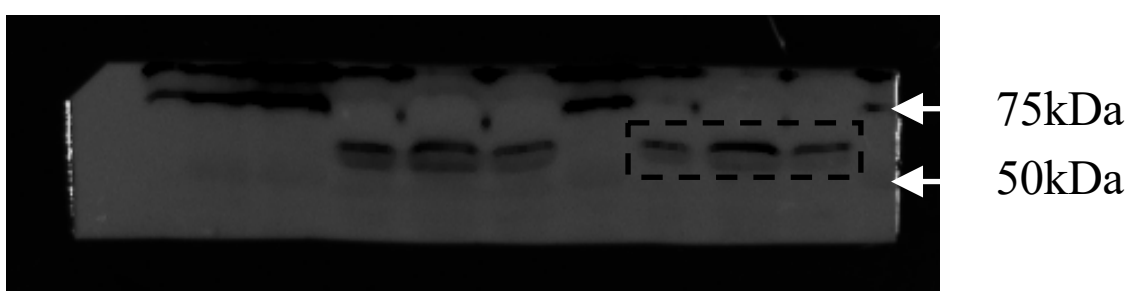

GAPDH (37 kDa)

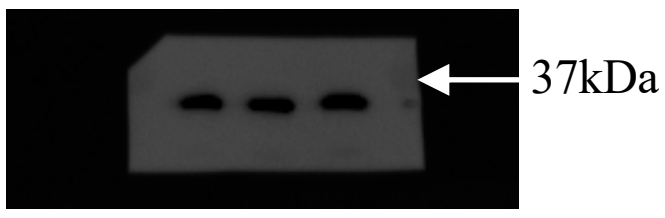

p21 (21 kDa)

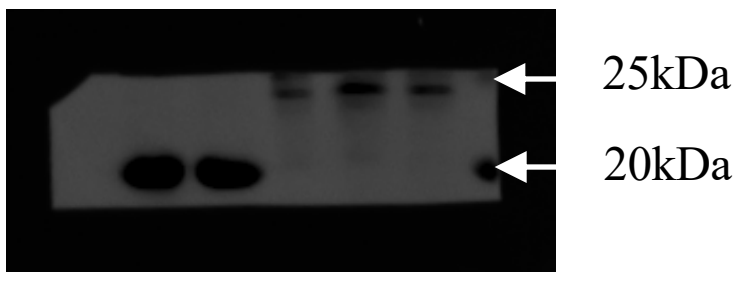

yH2A.X (15 kDa)

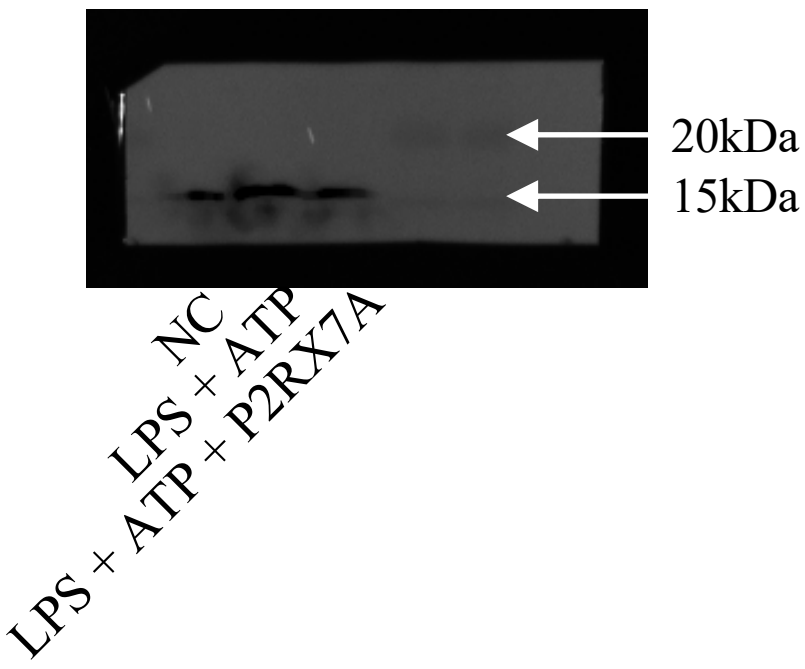

Figure S8C
P2RX7 (72 kDa)

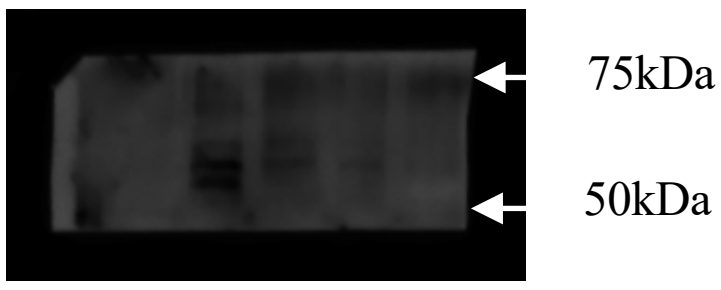

GAPDH (37 kDa)

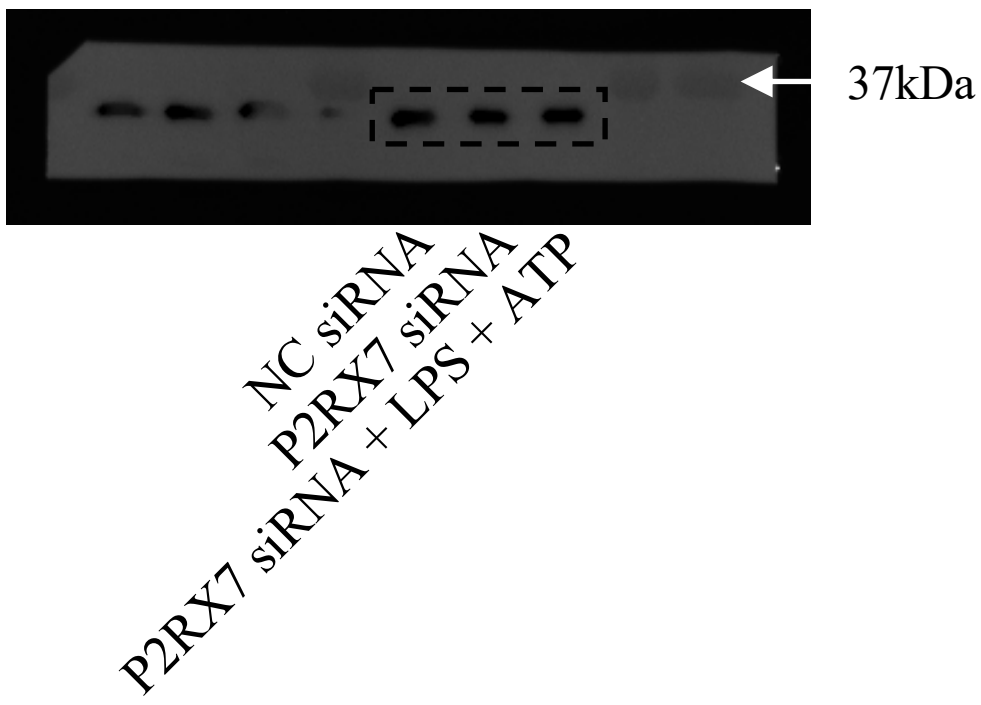

Figure S8E
p-Rb (110 kDa)

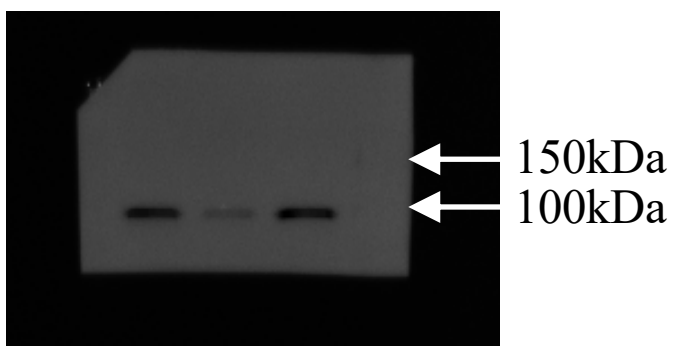

Rb (110 kDa)

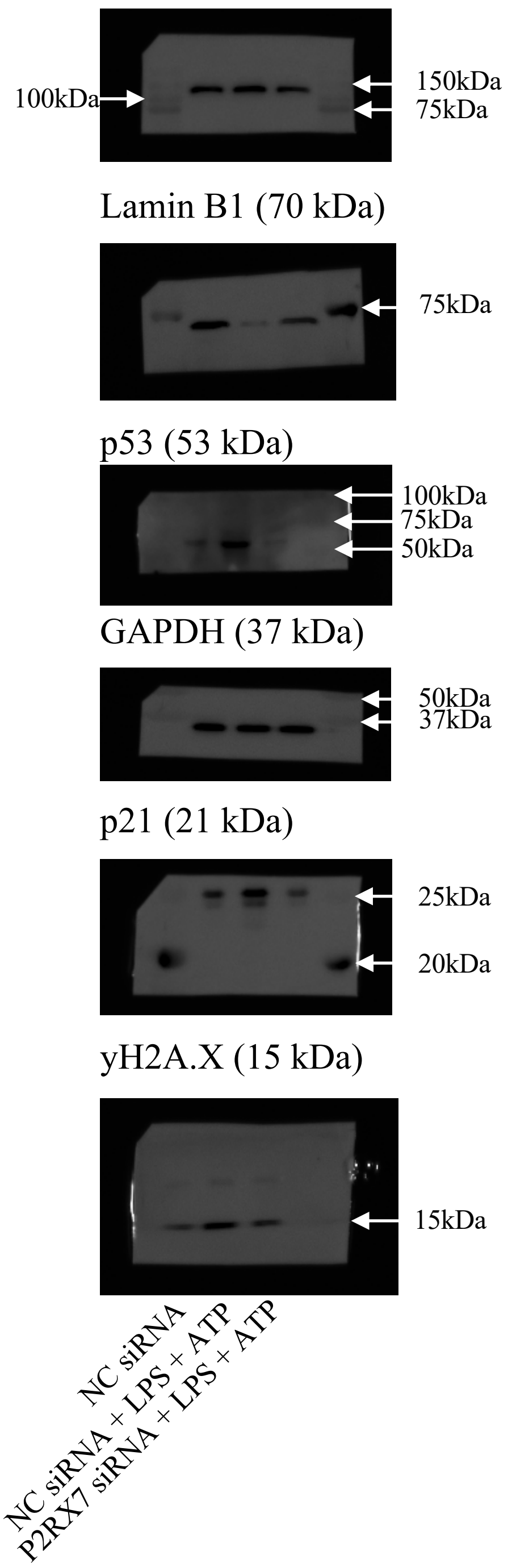
100kDa
150kDa
75kDa
Lamin B1 (70 kDa)
75kDa
p53 (53 kDa)
100kDa
75kDa
50kDa
GAPDH (37 kDa)
50kDa
37kDa
p21 (21 kDa)
25kDa
20kDa
yH2A.X (15 kDa)
15kDa
NC siRNA
NC siRNA + LPS + ATP
P2RX7 siRNA + LPS + ATP
